# Shielded bifunctional nanoreactor enabled tandem catalysis for plasma methane coupling

**DOI:** 10.1038/s41467-025-59709-y

**Published:** 2025-05-17

**Authors:** Chunqiang Lu, Yaolin Wang, Dong Tian, Ruidong Xu, Roong Jien Wong, Shibo Xi, Wen Liu, Hua Wang, Xin Tu, Kongzhai Li

**Affiliations:** 1 State Key Laboratory of Complex Nonferrous Metal Resources Clean Utilization, Kunming University of Science and Technology, Kunming, 650093 P. R. China; 2 Faculty of Metallurgical and Energy Engineering, Kunming University of Science and Technology, Kunming, 650093 P. R. China; 3 Department of Electrical Engineering and Electronics, University of Liverpool, Liverpool, L69 3GJ UK; 4 School of Chemistry, Chemical Engineering and Biotechnology, Nanyang Technological University, 62 Nanyang Drive, Singapore, 637459 Singapore; 5 Institute of Sustainability for Chemicals, Energy and Environment (ISCE2), Agency for Science, Technology and Research (A*STAR), 1 Pesek Road, Jurong Island, Singapore, 627833 Republic of Singapore; 6 Southwest United Graduate School, Kunming, 650092 P. R. China

**Keywords:** Chemical engineering, Heterogeneous catalysis, Nanoscale materials, Renewable energy

## Abstract

The direct conversion of methane into valuable unsaturated C_2_ hydrocarbons (C_2_H_2_ and C_2_H_4_) attracts growing attention. Non-thermal plasma offers a promising approach for this process under mild conditions. However, the competing formation of C_2_H_6_ and excessive dehydrogenation limit the selectivity toward C_2_H_2_ and C_2_H_4_. Herein, we develop a promising shielded bifunctional nanoreactor with a hollow structure and mesoporous channels (Na_2_WO_4_-Mn_3_O_4_/m-SiO_2_) that effectively limits CH_4_ overactivation and promotes selective coupling to form C_2_H_2_ and C_2_H_4_ under plasma activation, achieving 39% CH_4_ conversion with 42.3% C_2_H_2_ and C_2_H_4_ fraction. This nanoreactor features isolated Na_2_WO_4_ embedded within the channels and Mn_3_O_4_ confined in the cavity of the SiO_2_ hollow nanospheres, enabling internal tandem catalysis at co-located active sites. Na_2_WO_4_ induces the conversion of diffused CH_4_ and CH_3_ into reactive intermediates (^*^CH and ^*^CH_2_), which subsequently couple on the Mn_3_O_4_ surface to form C_2_H_2_ and C_2_H_4_. Furthermore, the mesoporous channels inhibit the plasma discharge within the nanoreactor, preventing deep dehydrogenation of CH_x_ species to solid carbon. This nanoreactor demonstrates a highly selective route for the nonoxidative conversion of methane to valuable C_2_ hydrocarbons, offering a new paradigm for the rational design of catalysts for plasma-driven chemical processes.

## Abstract (web summary)

Non-thermal plasma enables direct methane conversion to valuable C_2_ hydrocarbons, but selectivity is hindered by C_2_H_6_ formation and over-dehydrogenation. Here, the authors present a shielded bifunctional nanoreactor with a hollow, mesoporous design that curbs CH_4_ overactivation and enhances selective C_2_H_2_ and C_2_H_4_ production under plasma activation.

## Introduction

Unsaturated C_2_ hydrocarbons, crucial building blocks in the chemical industry, are primarily produced from the energy-intensive processing of crude oil. The development of sustainable alternatives via gas conversion has attracted increasing interest^1,2^. While conventional gas conversion methods rely on indirect syngas routes, direct methane conversion to unsaturated C_2_ hydrocarbons offers a more attractive pathway^3,4^. Direct conversion of CH_4_ to value-added fuels and chemicals can be achieved through either nonoxidative or oxidative catalytic routes^5–7^. The nonoxidative pathway offers high C_2_ selectivity and atom utilization efficiency but demands endothermic conditions (up to 1100 °C), incurring substantial energy costs and CO_2_ emissions^1^. In contrast, oxidative coupling of methane (OCM) can significantly lower the reaction temperature to ~600 °C in the presence of O_2_^8,9^. However, this method suffers from overoxidation of CH_4_ to thermodynamically favored CO and CO_2_, limiting the yield of unsaturated C_2_ hydrocarbons^8,10^. These challenges highlight the urgent need for innovative strategies to enhance methane activation and coupling efficiency.

Non-thermal plasma (NTP) offers a promising solution for the homolytic activation of CH_4_ into radicals, enabling nonoxidative coupling of methane (NOCM) under mild conditions^11,12^. Among NTP techniques, dielectric barrier discharge (DBD) has been extensively explored for methane coupling. However, the main challenge is the limited control over product distribution, particularly in achieving selective production of unsaturated C_2_ hydrocarbons, such as acetylene (C_2_H_2_) and ethylene (C_2_H_4_). Instead, ethane (C_2_H_6_) is predominantly produced in NOCM using DBD reactors^11,13–16^. This selectivity issue originates from the significantly longer lifetime of CH_3_ radicals (>1 ms) compared to CH_2_ (<30 ns) and CH (<5 ns) radicals, which favors undesired reaction pathways^17^. Integrating tailored catalysts into DBD reactors has great potential to enhance selectivity toward targeted products^14,18,19^. Ideally, such catalysts would regulate the dehydrogenation process and selectively stabilize CH and CH_2_ intermediates, directing the reaction toward the formation of C_2_H_2_ and C_2_H_4_.

The plasma-catalytic NOCM process involves heterogeneous surface reactions, highlighting the importance of the rational design of catalysts with tailored active sites and morphologies to maximize performance. In conventional OCM, Na_2_WO_4_ and Mn_2_O_3_ have proven effective in CH_4_ activation and C-C coupling^20^. Specifically, Na_2_WO_4_ generates reactive oxygen species to activate CH_4_, while Mn_2_O_3_-supported O species promote the coupling of ^*^CH_3_ intermediates^21–23^. Given their critical roles in methane coupling, integrating Na_2_WO_4_ and Mn_2_O_3_ into plasma-catalytic NOCM offers a promising avenue to enhance the production of unsaturated C_2_ hydrocarbons through their synergistic effects – a strategy that remains underexplored. Notably, Na_2_WO_4_ and Mn_2_O_3_ function at distinct stages of the methane coupling pathway. Plasma pre-activates CH_4_, facilitating Na_2_WO_4_-mediated enhancement of CH_x_ dehydrogenation into ^*^CH and ^*^CH_2_ surface species, while Mn_2_O_3_ promotes subsequent coupling of these intermediates to form C_2_H_2_ and C_2_H_4_. Inspired by this synergistic interplay, we propose that a tandem plasma-catalysis system using spatially structured Na_2_WO_4_-MnO_x_ catalysts—designed to sequentially optimize dehydrogenation and coupling steps—could potentially achieve enhanced efficiency in C_2_ hydrocarbon formation compared to plasma-catalysis systems using randomly structured catalysts.

To address the challenges of overactivation and selectivity control in plasma-assisted methane coupling, we propose a promising assembled nanoreactor (Na_2_WO_4_-Mn_3_O_4_/m-SiO_2_, denoted as WMO/m-SiO_2_) designed for integration into a DBD plasma reactor (Supplementary Fig. 1). This nanoreactor features a hollow structure with mesoporous channels, accessibility to reactants both internally and externally. This design provides a shielding effect for CH_4_ molecules within the channels, preventing excessive dehydrogenation by plasma-generated reactive species. Furthermore, the WMO/m-SiO_2_ reactor demonstrates tandem catalytic functionality, significantly enhancing selectivity toward target C_2_ products. Specifically, Na_2_WO_4_ located within the m-SiO_2_ channels initiates CH_4_ activation, generating ^*^CH_2_ and ^*^CH surface intermediates. Concurrently, Mn_3_O_4_ species confined within the cavity of the SiO_2_ spheres promote facile coupling of these CH and CH_2_ intermediates to form C_2_H_2_ and C_2_H_4_. Our study demonstrates significantly enhanced C_2_H_2_ and C_2_H_4_ yields compared to conventional catalysts under analogous DBD plasma conditions. This work offers a strategy to overcome critical limitations in plasma-catalysis, advancing efficient methane conversion to high-value C_2_ hydrocarbons.

## Results

### Structural characterization of the catalysts

**Fig. 1:**
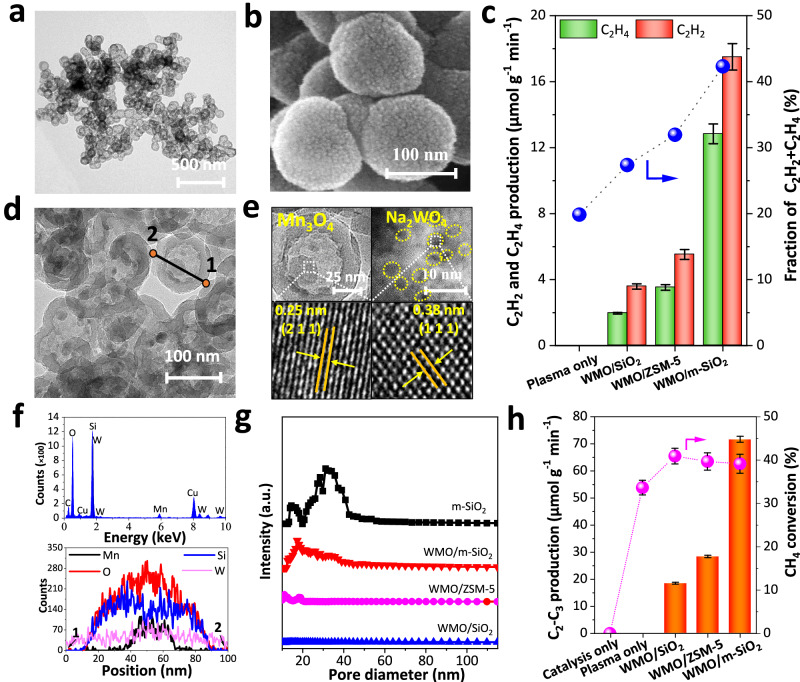
Characterization and catalytic performance. **a** TEM image and particle size distribution of m-SiO_2_. **b** SEM image of WMO/m-SiO_2_. **c** Production rate and molar fraction of C_2_H_4_ and C_2_H_2_ within C_2_-C_3_ hydrocarbons (Conditions: 1 bar, specific energy input (SEI) 5.1 kJ L^−1^, where SEI is defined as plasma discharge power divided by the gas flow rate; Feed gas 5 vol% CH_4_/Ar, total flow rate 200 mL min^−1^, discharge power 17 W, experiment duration 60 min). Error bars (standard deviation) in the figure were obtained from three sampling runs. **d**, **e** TEM and HRTEM images of WMO/m-SiO_2_. **f** EDS line scans (from Point 1 to Point 2) from Fig. 1d. **g** Pore size distributions of m-SiO_2_, WMO/m-SiO_2_, WMO/ZSM-5, and WMO/SiO_2_. **h** Production rate of C_2_-C_3_ hydrocarbons and methane conversion for catalysis-only, plasma-only and plasma-catalysis systems.

The synthesized mesoporous SiO_2_ (m-SiO_2_) featured interconnected nanospheres with diameters ranging from 95 to 135 nm (Fig. 1a and Supplementary Fig. 2a). Each nanosphere exhibited a hollow structure with a cavity diameter of ~65 nm and a shell thickness of ~20 nm (Supplementary Fig. 2b), along with a relatively high specific surface area of 240 m^2^ g^−1^ (Supplementary Table 1). Loading manganese and tungsten species onto m-SiO_2_ did not affect the hollow structure, and the resulting WMO/m-SiO_2_ nanoreactor retained surface porosity with particles localized both within the cavity and on the external surface (Fig. 1b, d and Supplementary Fig. 3). X-ray diffraction (XRD) confirmed the presence of Mn_3_O_4_ and amorphous SiO_2_ in the WMO/m-SiO_2_ composite (Supplementary Fig. 4). High-resolution transmission electron microscopy (HRTEM) revealed that the dispersed particles on the shell and cavity walls of m-SiO_2_ were Na_2_WO_4_ and Mn_3_O_4_, respectively (Fig. 1d–f and Supplementary Fig. 3). The measured lattice spacings of 0.38 and 0.25 nm corresponded to Na_2_WO_4_ (111) and Mn_3_O_4_ (211), respectively (Fig. 1e). Notably, although XRD confirmed the presence of Mn_3_O_4_ (Supplementary Fig. 4), scanning electron microscopy (SEM) elemental distribution mapping (Supplementary Figs. 5, 6) of WMO/m-SiO_2_ revealed negligible Mn and W signals compared to WMO/SiO_2_ and WMO/ZSM-5 (Zeolite Socony Mobil-5). Unless otherwise specified, WMO/m-SiO_2_, WMO/SiO_2_, and WMO/ZSM-5 refer to samples with 1% Na_2_WO_4_-5% Mn loaded on the support material. These findings indicate that Na_2_WO_4_ and Mn_3_O_4_ particles are predominantly encapsulated within the m-SiO_2_ spheres. Additionally, energy-dispersive X-ray spectroscopy (EDS) scans in Fig. 1f further confirmed the presence of Mn species inside the m-SiO_2_ cavity.

### Plasma-catalytic NOCM reaction

We evaluated CH_4_ conversion under three different conditions: plasma-only (no catalyst, no external heating), catalysis-only (external heating at 250 °C, no plasma), and plasma-catalysis (coupled plasma and catalysts, no external heating). Under plasma-only and plasma-catalysis conditions, the measured temperature was ~250 °C. In plasma-only mode, C_2_-C_3_ hydrocarbons dominated, with a maximum production of 31.2 μmol min^−1^ at a CH_4_ conversion of 33% (Supplementary Figs. 7, 8). Hydrocarbons with four or more carbon atoms were excluded due to negligible concentrations (<5% relative selectivity compared to C_2_ products). Figure 1c shows the distributions of C_2_H_4_ and C_2_H_2_ within the C_2_-C_3_ range. A synchronized increase in C_2_H_2_ and C_2_H_4_ proportions was observed, which can be attributed to the closely aligned energetic thresholds of electron-induced CH_4_ conversion into CH_2_ and CH species^13^. WMO/m-SiO_2_ exhibited no catalytic activity for methane conversion at 250 °C in the absence of plasma. However, under NTP conditions with WMO/m-SiO_2_, the production of C_2_H_4_ and C_2_H_2_ significantly increased to 30.3 μmol g^−1^ min^−1^ (12.8 μmol g^−1^ min^−1^ for C_2_H_4_ and 17.5 μmol g^−1^ min^−1^ for C_2_H_2_), surpassing WMO/SiO_2_ and WMO/ZSM-5 by factors of ~5 and 3.4, respectively (Fig. 1c). With the WMO/m-SiO_2_ nanoreactor, the proportion of unsaturated hydrocarbons in the C_2_–C_3_ range increased significantly from 17.7% (plasma-only) to 42.3% (Fig. 1c and Supplementary Fig. 9). Simultaneously, the total yield of C_2_H_4_ increased markedly from 2.6 to 6.4 μmol min^−1^, while C_2_H_2_ increased from 3.1 to 8.8 μmol min^−1^. Notably, all three catalysts selectively promoted C_2_H_4_ and C_2_H_2_ production via deep dehydrogenation and coupling of CH_4_, rather than promoting overall methane conversion (Fig. 1h).

The pore sizes of m-SiO_2_ and WMO/m-SiO_2_ ranged from 20 to 40 nm, distinct from those of WMO/SiO_2_ and WMO/ZSM-5 (Fig. 1g and Supplementary Fig. 10). As shown in Supplementary Table 1, WMO/ZSM-5 exhibited the highest surface area of 226 m^2^ g^−1^, significantly exceeding that of WMO/m-SiO_2_ (67 m^2^ g^−1^). However, plasma-only and plasma-catalysis conditions (using WMO/ZSM-5, WMO/SiO_2_ and WMO/m-SiO_2_) exhibited similar discharge properties (Supplementary Figs. 11–13). Additionally, packing the discharge zone with silica supports yielded consistent C_2_H_2_ and C_2_H_4_ levels (Supplementary Fig. 14). Furthermore, despite its lower surface area, WMO/m-SiO_2_ demonstrated better CH_4_ coupling efficiency to C_2_H_2_ and C_2_H_4_, highlighting the critical role of its unique hollow mesoporous structure in enhancing the synergistic interaction between NTP and catalysis.

### Evaluation of the effect of catalyst position on performance

**Fig. 2:**
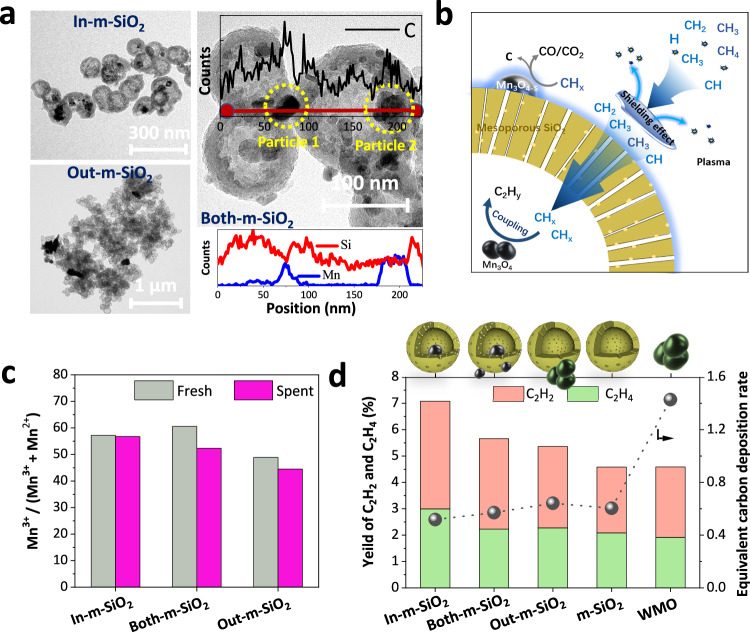
Effect of catalyst location m-SiO_2_ on NTP-CM performance. **a** TEM image and EDS spectra of spent catalysts In-m-SiO_2_, Out-m-SiO_2_, and Both-m-SiO_2_ (after 60 min of reaction). **b** Schematic illustration of the role of m-SiO_2_ and the effect of catalyst position on methane coupling. **c** Mn^3+^/(Mn^3+^+Mn^2+^) ratio for fresh and spent catalysts. **d** Yields of C_2_H_2_ and C_2_H_4_ (defined as the product of CH_4_ conversion and the selectivity of C_2_H_2_ and C_2_H_4_) and equivalent carbon deposition rate (ECR, defined as the solid carbon selectivity on the catalyst divided by the methane conversion) (Conditions: 1 bar, SEI 5.1 kJ L^−1^, total flow rate 200 mL min^−1^, discharge power 17 W, experiment duration 60 min).

TEM and scanning transmission electron microscopy (STEM) images depict the morphology of the catalysts and the location of metal oxide particles, respectively (Fig. 2a and Supplementary Fig. 15). The metal oxide particles were selectively deposited in three configurations: (1) exclusively inside m-SiO_2_ (In-m-SiO_2_), (2) partially distributed within m-SiO_2_ (Both-m-SiO_2_), and (3) predominantly deposited on the exterior of m-SiO_2_ (Out-m-SiO_2_). For both “In-m-SiO_2_” and “Both-m-SiO_2_”, the Mn_3_O_4_ sizes ranged from 5 to 35 nm (Supplementary Fig. 15e, f). TEM analysis of Both-m-SiO_2_ revealed a significant reduction in the Si signal around Particle 1 (50–90 nm), while no similar decrease was observed near Particle 2 (150–200 nm) (Fig. 2a). Moreover, the Mn signal intensified in both particle types, suggesting that Particle 1 and Particle 2 are located on the exterior and interior surfaces of the m-SiO_2_ nanosphere, respectively (Fig. 2a). XRD patterns further confirmed the spatial distribution of Na_2_WO_4_ and Mn_3_O_4_ particles. Samples with higher diffraction intensities and more pronounced peaks corresponded to increased exposure of Mn_3_O_4_ species on the external surface of m-SiO_2_ (Supplementary Fig. 16).

EDS analysis of Particle 1 (internal) revealed weaker carbon and stronger oxygen signals compared to Particle 2 (external) (Fig. 2a and Supplementary Fig. 17). This suggests that m-SiO_2_ protects Mn_3_O_4_ particles from direct exposure to CH_4_ plasma. This shielding effect arises from the “Debye shielding” mechanism, where plasma discharge cannot penetrate the mesopores of m-SiO_2_ due to their pore diameters being smaller than the Debye length (typically hundreds of nanometers)^19,24,25^. Thus, the shielded internal cavity prevents the reduction of Mn_3_O_4_ particles and mitigates direct carbon deposition. This hypothesis is further supported by the observed decrease in the Mn^3+^/(Mn^3+^+Mn^2+^) ratio as more manganese oxide particles are located outside the m-SiO_2_ layer (Fig. 2c and Supplementary Fig. 18). Previous studies have shown that low-valent metal catalysts (oxides, carbides, or metals) are highly effective for the deep dehydrogenation of CH_4_^26,27^. Consistent with these findings, Supplementary Fig. 19 shows that Mn_3_O_4_ particles exhibited a stronger carbon signal than the m-SiO_2_ surface. Furthermore, as Na_2_WO_4_ and Mn_3_O_4_ particles are encapsulated within m-SiO_2_ nanospheres, the yield of C_2_H_2_ and C_2_H_4_ decreased from 7.0% (WMO/m-SiO_2_) to 4.5% (Na_2_WO_4_-Mn_3_O_4_, denoted as WMO). This trend implies that converting an equivalent amount of methane leads to more carbon deposition when fewer encapsulated Na_2_WO_4_ and Mn_3_O_4_ particles are present (Fig. 2d and Supplementary Fig. 20).

### The role of Na_2_WO_4_ and Mn_3_O_4_ in plasma-catalytic NOCM reaction

**Fig. 3:**
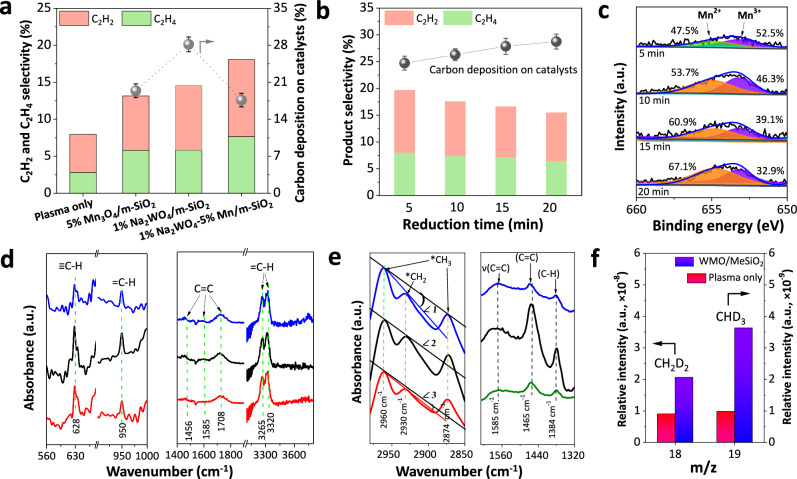
Performance of Mn and W species. **a** Selectivity of products (carbon deposited on the catalyst, C_2_H_4_ and C_2_H_2_) for plasma-only and plasma-catalysis systems (Conditions: 1 bar, SEI 5.1 kJ L^−1^, total flow rate 200 mL min^−1^, discharge power 17 W, experiment duration 60 min). Error bars (standard deviation) in the figure were obtained from three sampling runs. **b** Selectivity of carbon deposited on the catalyst and selectivity of C_2_H_4_ and C_2_H_2_ for reduced WMO/m-SiO_2_ (reduced at 450 °C with H_2_). **c** Mn 2*p* XPS spectra of reduced WMO/m-SiO_2_ (pretreated with H_2_ for 5, 10, 20, and 25 min). **d** IR spectra of WMO/m-SiO_2_ (black), Mn_3_O_4_/m-SiO_2_ (blue), and Na_2_WO_4_/m-SiO_2_ (red) under plasma-catalysis conditions. **e** Quasi-in situ DRIFT spectra of catalysts after plasma-catalytic NOCM reaction. **f** CH_2_D_2_ and C_2_HD_3_ species generated under plasma-only and plasma-catalysis (with WMO/m-SiO_2_) conditions (feed gas 2.5 vol% CH_4_-2.5 vol% CD_4_/Ar, SEI 5.1 kJ L^−1^, total flow rate 200 mL min^−1^; experiment duration 60 min).

The 1% Na_2_WO_4_-5% Mn_3_O_4_/m-SiO_2_ catalyst achieved a combined C_2_H_4_ and C_2_H_2_ selectivity of 18.1%, surpassing that of Na_2_WO_4_/m-SiO_2_ (14.5%), 5% Mn_3_O_4_/m-SiO_2_ (13.2%) and plasma-only conditions (7.9%) (Fig. 3a and Supplementary Fig. 21). A comparison of CH_4_ conversion and C_2_-C_3_ hydrocarbon distribution in the DBD reactor with previous studies is provided in Supplementary Table 2. The 1% Na_2_WO_4_-5% Mn_3_O_4_/m-SiO_2_ (WMO/m-SiO_2_) catalyst demonstrated the high selectivity for C_2_H_2_ and C_2_H_4_, while maintaining competitive methane conversion. Among reported studies, this work achieved a lower energy cost (EC) for CH_4_ conversion (6.8 MJ/mol), demonstrating the effectiveness of the catalyst in plasma-catalytic NOCM reaction. Notably, the catalyst exhibited stable performance for over 25 h (Supplementary Figs. 22, 23). X-ray photoelectron spectroscopy (XPS) and XRD analyses confirmed that the dominant oxidation states of tungsten and manganese species in WMO/m-SiO_2_, as well as in 5% Mn/m-SiO_2_ and 1% Na_2_WO_4_/m-SiO_2_, remained unchanged after the reaction (Supplementary Figs. 24–26). Supplementary Fig. 21 shows that increasing the Na_2_WO_4_ loading from 0.5 to 5% increased carbon deposition from 16.4 to 24.6%. In contrast, higher Mn_3_O_4_ content reduced carbon deposition. These findings suggest that Na_2_WO_4_/m-SiO_2_ more effectively promotes the further dehydrogenation of methane compared to Mn_3_O_4_/m-SiO_2_.

The involvement of catalyst-bound oxygen species is well established in methane dehydrogenation^5,9^ and the coupling of intermediate species^28–30^, highlighting their crucial roles in these reactions. To investigate this effect, we pretreated WMO/m-SiO_2_ with H_2_ at 450 °C for different durations, generating a series of WMO/m-SiO_2_ samples with varying oxygen contents. H_2_ pretreatment at 450 °C primarily reduced Mn_3_O_4_ but not tungsten species, as evidenced by H_2_ temperature-programmed reduction (H_2_-TPR) and XPS (Fig. 3c and Supplementary Figs. 27, 28). H_2_ treatment resulted in increased carbon deposition and decreased selectivity for C_2_H_4_ and C_2_H_2_ when the WMO/m-SiO_2_ nanoreactor was reduced for 5–20 min, accompanied by a decline in Mn^3+^ content and oxygen loading within the catalyst (Fig. 3b, c). These observations align with the trend that increasing the Mn content (β) from 2 to 10% in 1% Na_2_WO_4_-β Mn_3_O_4_/m-SiO_2_ slightly enhanced the production of C_2_-C_3_ hydrocarbons (Supplementary Fig. 29). These findings suggest that MnO_x_ acts as an oxygen carrier, promoting the coupling of active species (CH_x_ and H) and reducing methane cracking. This highlights the importance of optimizing oxygen content in catalysts to maximize performance.

### CH_x_ species absorbed on the catalyst and in the gas phase

In situ plasma-coupled Fourier transform infrared (FTIR) spectroscopy was used to investigate plasma-assisted surface reactions on WMO/m-SiO_2_, 5% Mn_3_O_4_/m-SiO_2_ and 1% Na_2_WO_4_/m-SiO_2_. As shown in Supplementary Fig. 30, the intensities of the IR peaks at 628 cm^−1^ (≡C-H) and 950 cm^−1^ (=C-H) decreased as the reaction progressed. These absorbed ≡C-H and =C-H bands are associated with key intermediates involved in the formation of C_2_H_2_ and C_2_H_4_ during the catalytic process^31^. Additional IR bands at 1465, 1585, 1708, 3265, and 3320 cm^−1^ correspond to C = C stretching vibrations on the catalyst surface (Fig. 3d)^31–33^. Notably, WMO/m-SiO_2_ exhibited the highest intensities for peaks associated with ≡C-H, =C-H, and C=C compared to catalysts containing only Mn or W. This finding suggests that the synergistic interaction between Mn_3_O_4_ and Na_2_WO_4_ sites effectively promotes the formation of surface-adsorbed ^*^CH and ^*^CH_2_ groups, ultimately enhancing the yield of C_2_H_4_ and C_2_H_2_.

Quasi-in situ DRIFTS characterization provided further insights into the types of adsorbed species remaining on the catalyst surface after the reaction (Supplementary Figs. 31, 32). As shown in Fig. 3e, several key peaks were observed, including C–H stretching from ^*^CH_3_ (2960 and 2872 cm^−1^)^34^, C–H stretching from ^*^CH_2_ (2930 cm^−1^)^34^, C=C bonds (1585 and 1465 cm^−1^)^32,33^ and C-H bond bending/deformation modes (1384 cm^−1^)^35^. In this study, the angles (∠1 = 12°, ∠2 = 0°, and ∠3 = 3°) between the standard slope and tangents (peak 2960 to peak 2930 cm^−1^) were used to measure the relative proportions of absorbed ^*^CH_3_ and ^*^CH_2_ species on the catalyst surface. These species serve as precursors for the formation of ethane and ethylene, respectively^36^. Among the catalysts, 1% Na_2_WO_4_-5% Mn_3_O_4_/m-SiO_2_ exhibited the highest ^*^CH_2_ intensity, followed by 1% Na_2_WO_4_/m-SiO_2_ and 5% Mn/m-SiO_2_. Notably, compared to 1% Na_2_WO_4_/m-SiO_2_, 5% Mn/m-SiO_2_ promoted the formation of surface C=C (1585 and 1465 cm^−1^), indicating that ^*^CH_2_ formation predominantly occurred at W sites, while Mn sites facilitated the coupling of CH_2_ to form C=C bonds. Although the internal Mn_3_O_4_ sites are not directly exposed to plasma (Supplementary Fig. 3), varying the Mn_3_O_4_ loading within the m-SiO_2_ spheres led to observable changes in product distribution and the relative intensity of adsorbed ^*^CH_3_ and ^*^CH_2_ species (Supplementary Figs. 21, 31). This suggests that CH_x_ radicals can diffuse or migrate at least 20 nm to reach Mn_3_O_4_ sites within their lifetime, enabling them to access the interior of the catalyst for subsequent reactions.

### Methane isotopic labeling experiments

Methane isotopic labeling experiments, conducted using a parallel flow of CH_4_ and CD_4_, revealed an increase in CH_3_ and CH_2_ radicals during the plasma-catalyzed reaction over WMO/m-SiO_2_ compared to the plasma-only condition (Fig. 3f). Detailed experimental procedures are provided in the Methods section, and the corresponding conversions of CD_4_ and CH_4_, along with product distributions, are shown in Supplementary Fig. 33. The experiment was designed to probe the complex dynamics within the discharge field, where multiple collisions and coupling reactions occur, leading to the reversible activation of C-H bonds, facilitating the reformation of nascent methane from activated C_x_H_y_ intermediates^36^. This behavior contrasts with the essentially irreversible C-H activation in traditional OCM^29^. The detection of mixed CH_2_D_2_ and CHD_3_ isotopes during plasma-catalyzed NOCM on WMO/m-SiO_2_ confirmed this mechanism. Compared to the plasma-only system, the plasma-catalytic system with WMO/m-SiO_2_ demonstrated significantly higher production of CH_2_D_2_ and CHD_3_ (Fig. 3f). This result suggests that WMO/m-SiO_2_ enhances the activation of CH_4_ and the generation of CH_3_/CD_3_ and CH_2_/CD_2_ radicals. The enriched pool of CH_2_ species ultimately promotes dimerization into C_2_H_2_ and C_2_H_4_^11^. In summary, the synergistic interaction between Mn_3_O_4_ and Na_2_WO_4_ sites in the WMO/m-SiO_2_ nanoreactor effectively promotes the formation of surface-adsorbed ^*^CH and ^*^CH_2_ species, as well as gas-phase CH_2_ radicals, leading to enhanced yields of C_2_H_2_ and C_2_H_4_.

### Density functional theory (DFT) calculations

**Fig. 4:**
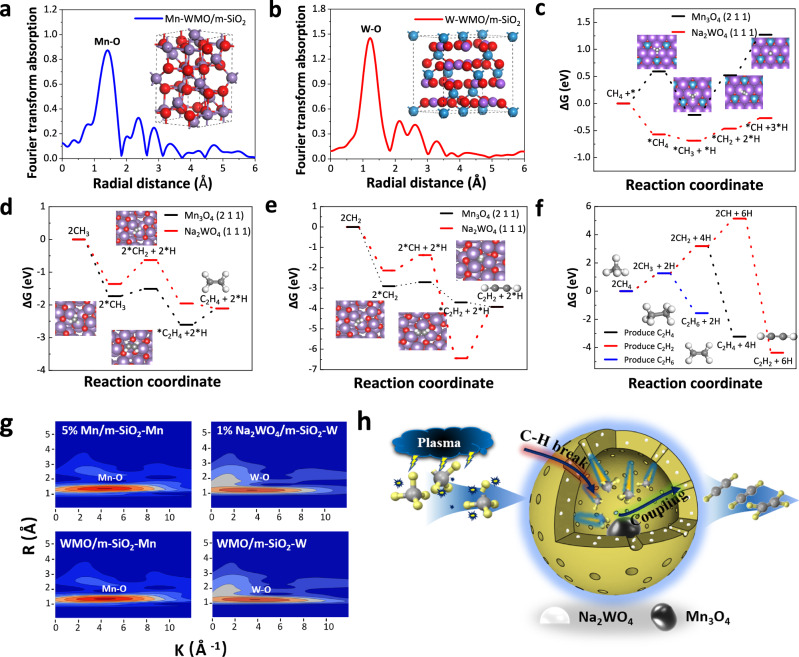
The structures of WMO/m-SiO_2_ and DFT calculations. **a** Fourier transform (FT) of the EXAFS spectrum of Mn and the DFT-optimized structure of Mn_3_O_4_ on WMO/m-SiO_2_. **b** FT-EXAFS spectra of W and DFT-optimized structure of Na_2_WO_4_ on WMO/m-SiO_2_. **c** DFT-optimized geometries of ^*^CH_4_ dehydrogenation to ^*^CH_3_, ^*^CH_2_, and ^*^CH on Mn_3_O_4_ (211) and Na_2_WO_4_ (111) surfaces. **d** DFT-optimized geometries of ^*^CH_3_ to C_2_H_4_ on Mn_3_O_4_ (211) and Na_2_WO_4_ (111) surfaces. **e** DFT-optimized geometries of ^*^CH_2_ coupled to C_2_H_2_ on Mn_3_O_4_ (211) and Na_2_WO_4_ (111) surfaces. **f** DFT-optimized geometries of intermediates in the plasma-catalytic conversion of CH_4_ to radicals, C_2_H_2_, C_2_H_4_, and C_2_H_6_. **g** Wavelet transform plots of the Mn K-edge and W K-edge. **h** Schematic illustration of C_2_H_2_ and C_2_H_4_ formation pathways on WMO/m-SiO_2_.

Synchrotron radiation-based X-ray absorption spectroscopy (XAS) was employed to elucidate the chemical state and local structure of the WMO/m-SiO_2_ catalysts. The X-ray absorption near-edge structure (XANES) spectra at the Mn K-edge (Mn_3_O_4_/m-SiO_2_) and W K-edge (Na_2_WO_4_/m-SiO_2_) in WMO/m-SiO_2_ closely resembled those of the individual Mn_3_O_4_/m-SiO_2_ and Na_2_WO_4_/m-SiO_2_ reference catalysts (Supplementary Fig. 34). This observation, in substantial contrast to the combined Na_2_WO_4_-Mn_3_O_4_ spectrum, strongly suggests the presence of isolated Mn_3_O_4_ and Na_2_WO_4_ active sites dispersed on the m-SiO_2_ support. Further validation was provided by Fourier transform (FT) extended X-ray absorption fine structure spectroscopy (EXAFS) results (Fig. 4a, b). The peaks at 1.4 Å (Fig. 4a) and 1.2 Å (Fig. 4b) mainly represent the single scattering of Mn−O and W–O bonds, respectively, confirming the existence of isolated Mn and W species and the absence of direct Mn-W binding in the WMO/m-SiO_2_ catalyst. The bond lengths for Mn–O and W–O in WMO/m-SiO_2_ were determined to be 1.86 and 1.69 Å, respectively (Supplementary Table 3). The proposed Mn-O model exhibited excellent agreement with the experimental spectra, as evidenced by its superior fit in the XANES analysis and negligible deviations from DFT calculations (Supplementary Figs. 34–36).

To elucidate the fundamental mechanism of C_2_H_2_ and C_2_H_4_ production within WMO/m-SiO_2_, spin-polarized periodic DFT calculations were employed to unravel the distinct roles of its components during plasma-catalytic NOCM reaction. Based on experimental results (Fig. 1e and Supplementary Fig. 3), we selected Mn_3_O_4_ (211) and Na_2_WO_4_ (111) surfaces as model systems (Supplementary Fig. 35). The calculated binding energies of reaction intermediates involved in CH_4_ dehydrogenation and subsequent coupling to C_2_H_2_ and C_2_H_4_ are presented in Supplementary Tables 4, 5. Notably, the binding strength of the C_x_H_y_ intermediates followed the order of Mn_3_O_4_ (211) > Na_2_WO_4_ (111), with intermediates binding via C or C-C on both surfaces (Supplementary Figs. 37, 38 and Supplementary Tables S4, S5).

The calculated Gibbs free energy change profiles for CH_4_ dehydrogenation and intermediate active species coupling on the Mn_3_O_4_ (211) and Na_2_WO_4_ (111) catalysts are shown in Fig. 4c–e. For CH_4_ dehydrogenation (Fig. 4c), the Na_2_WO_4_ (111) surface can facilitate CH_4_ dehydrogenation to produce ^*^CH_3_, ^*^CH_2_, and ^*^CH species. The most challenging step was ^*^CH_3_ dehydrogenation, with an uphill energy of 0.22 eV, followed by ^*^CH_2_ dehydrogenation, with an uphill energy of 0.19 eV on the Na_2_WO_4_ (111) surface. In contrast, CH_4_ dehydrogenation on the Mn_3_O_4_ (211) surface required significantly higher uphill energy (0.73 and 0.75 eV) for producing ^*^CH_2_ and ^*^CH species, respectively. In addition, the reaction energy barriers for ^*^CH_3_ → ^*^CH_2_ + ^*^H and ^*^CH_2_ → ^*^CH + ^*^H were calculated (Supplementary Table 8). These two elementary reactions were identified as the rate-determining steps in CH_4_ dehydrogenation to generate ^*^CH_3_, ^*^CH_2_, and ^*^CH species on both surfaces (Fig. 4c), with energy barriers of 0.97 and 0.69 eV on Na_2_WO_4_ (111), significantly lower than those on Mn_3_O_4_ (211) (1.93 and 2.02 eV). This indicates that the Na_2_WO_4_(111) surface is more effective in facilitating CH_4_ dehydrogenation, consistent with experimental results (Supplementary Fig. 21). The Mn_3_O_4_ (211) surface was found to be more favorable for coupling ^*^CH_2_ and ^*^CH species to generate C_2_H_4_ and C_2_H_2_ (Fig. 4d, e). The most challenging steps were the desorption of ^*^C_2_H_4_ (Fig. 4d) and ^*^CH_2_ dehydrogenation (Fig. 4e), with uphill energies of 0.49 and 0.19 eV, respectively. In contrast, on the Na_2_WO_4_ (111) surface, the most difficult steps were ^*^CH_3_ dehydrogenation (Fig. 4d) and desorption of ^*^C_2_H_2_ (Fig. 4e), with significantly higher uphill energies of 0.73 and 2.52 eV, respectively. In addition, we investigated CH_4_ dehydrogenation and radical coupling reactions under plasma-only conditions (Fig. 4f). The results suggest that plasma-driven NOCM without a catalyst favors the production of C_2_H_6_, in stack contrast to plasma-catalyzed reactions.

To elucidate the role of SiO_2_ in the WMO/m-SiO_2_ nanoreactor, SiO_2_/Mn_3_O_4_ (211) and SiO_2_/Na_2_WO_4_ (111) models were built (Supplementary Figs. 36, 39, 40). The calculated binding energies of reaction intermediates involved in CH_4_ dehydrogenation and subsequent coupling to form C_2_H_2_ and C_2_H_4_ are presented in Supplementary Tables 6 and 7, with the most stable adsorption configurations illustrated in Supplementary Figs. 39, 40. Notably, SiO_2_/Na_2_WO_4_ (111) exhibited significantly weaker binding for ^*^C_2_H_2_ compared to the isolated Na_2_WO_4_ (111) surface, with adsorption energies of −0.54 versus −0.95 eV, respectively (Supplementary Fig. 41 and Supplementary Tables 6, 7). In contrast, SiO_2_/Mn_3_O_4_ (211) favored the desorption of ^*^C_2_H_4_ compared to pure Mn_3_O_4_ (211), with an adsorption energy of −0.62 eV (compared to −1.58 eV on pure Na_2_WO_4_ (111)). The binding energies of ^*^CH/^*^CH_2_ on SiO_2_/Mn_3_O_4_ (211) and SiO_2_/Na_2_WO_4_ (111) were −6.75 and −5.77 eV and −2.61 and −5.43 eV, respectively, indicating that SiO_2_/Mn_3_O_4_ has a stronger adsorption capacity for ^*^CH_2_ and ^*^CH than SiO_2_/Na_2_WO_4_. In summary, DFT calculations reveal that Na_2_WO_4_ facilitates CH_4_ dehydrogenation to ^*^CH or ^*^CH_2_ intermediates, while Mn_3_O_4_ promotes the coupling of ^*^CH and ^*^CH_2_ to form C_2_H_2_ and C_2_H_4_, respectively. Furthermore, the presence of SiO_2_ in combination with Na_2_WO_4_ and Mn_3_O_4_ enhances the desorption of the generated ^*^C_2_H_2_ and ^*^C_2_H_4_ species. These findings are consistent with experimental observations.

### Reaction mechanisms

The enhanced catalytic performance of Na_2_WO_4_-Mn_3_O_4_/m-SiO_2_, compared to Na_2_WO_4_/m-SiO_2_ and Mn_3_O_4_/m-SiO_2_, highlights the synergistic effects between Na_2_WO_4_ and Mn_3_O_4_ sites (Supplementary Fig. 21). Quasi-in situ DRIFTS characterization (Fig. 3e and Supplementary Figs. 31, 32) indicates that W sites promote ^*^CH_2_ generation, while Mn sites facilitate the coupling of ^*^CH_2_ to form C=C bonds.

If radicals were fully converted at Na_2_WO_4_ before reaching Mn_3_O_4_ sites within m-SiO_2_, significant carbon deposition would be expected on Na_2_WO_4_. However, compared to Na_2_WO_4_/m-SiO_2_, the 1% Na_2_WO_4_-5% Mn_3_O_4_/m-SiO_2_ catalyst exhibits lower carbon accumulation (Supplementary Fig. 21) and higher ^*^CH_2_ and C=C intensities (Fig. 3e). These findings suggest that radicals initially generated on Na_2_WO_4_ sites undergo further transformation into ^*^CH and ^*^CH_2_, which subsequently migrate to Mn_3_O_4_ sites for coupling reactions to produce C_2_H_2_ and C_2_H_4_.

Wavelet transforms of the EXAFS spectra at the Mn K-edge and W K-edge for WMO/m-SiO_2_, 5% Mn_3_O_4_/m-SiO_2_, and 1% Na_2_WO_4_/m-SiO_2_ reveal similar Mn−O and W−O scattering peaks at (4.1, 1.4 Å) and (3.9, 1.2 Å), respectively (Fig. 4g). These findings indicate that Na_2_WO_4_ and Mn_3_O_4_ are independently distributed on the m-SiO_2_ support, consistent with TEM observations (Supplementary Fig. 3). Notably, TEM analysis also demonstrates the close spatial proximity of Na_2_WO_4_ and Mn_3_O_4_, providing potential pathways for intermediate species migration.

DFT calculations further reveal that the adsorption energies of ^*^CH and ^*^CH_2_ on Mn_3_O_4_ are −5.21 and −3.96 eV, respectively, significantly stronger than those on Na_2_WO_4_ (−3.27 and −3.08 eV) (Supplementary Tables 6, 7). Notably, when supported on m-SiO_2_, the adsorption energy gap increases, suggesting that radicals preferentially stabilize on Mn_3_O_4_ rather than remaining on Na_2_WO_4_ (Supplementary Fig. 41). This thermodynamic preference, combined with the close proximity of two active sites, indicates that ^*^CH and ^*^CH_2_ species likely undergo surface diffusion or desorption-reabsorption migration, facilitating C-C coupling on Mn_3_O_4_. Similar bifunctional catalysis mechanisms have been reported in thermal catalysis systems, where intermediate spillover between distinct active sites enhances reaction efficiency^37,38^.

The distribution and synergy between Na_2_WO_4_ and Mn_3_O_4_ sites further facilitate the sequential activation of C-H bonds and C-C coupling within the WMO/m-SiO_2_ nanoreactor. Herein, a tandem reaction mechanism is proposed for CH_4_ conversion to C_2_H_2_ and C_2_H_4_ (Fig. 3h): (I) CH_4_ dissociation and CH_3_ diffusion: Energetic electrons from the plasma induce CH_4_ dissociation into CH_x_ fragments, which subsequently diffuse toward the interior of the silica sphere due to the concentration gradient across m-SiO_2_. (II) Dehydrogenation at Na_2_WO_4_ sites (support on the channel): ^*^CH_3_ species undergo dehydrogenation at W sites, forming surface-adsorbed ^*^CH and ^*^CH_2_ species. (III) Surface species coupling on Mn sites: ^*^CH and ^*^CH_2_ species migrate from Na_2_WO_4_ to Mn_3_O_4_ sites, leading to C-C coupling for C_2_H_2_ and C_2_H_4_ production.

Notably, the mesoporous channels of m-SiO_2_ restrict plasma penetration into the interior of m-SiO_2_, thereby mitigating excessive CH_4_ activation (e.g., methane cracking) and suppressing carbon deposition. This structural confinement, combined with the synergistic tandem catalysis of Na_2_WO_4_ and Mn_3_O_4_, significantly enhances the yield of C_2_ products. Moreover, the shielding effect of the nanoreactor reduces plasma-induced product decomposition and recombination, further enhancing selectivity.

## Discussion

This work presents a promising nanoreactor catalyst design strategy that significantly improves the yield and selectivity of C_2_H_2_ and C_2_H_4_ through plasma-catalytic NOCM under mild conditions. The nanoreactor features a hollow nanosphere structure with Na_2_WO_4_ nanoparticles anchored on the interconnected channels and monodispersed Mn_3_O_4_ nanocrystals hosted within the internal cavity. By positioning the nanoreactors in the discharge area, methane conversion reached 34%, with a selectivity of 42.3% toward C_2_H_2_ and C_2_H_4_. This represents a nearly 4.5-fold increase in yield and a fourfold increase in selectivity for unsaturated C_2_ hydrocarbons compared to the plasma-only system. Importantly, no deactivation was observed during the 25-h catalyst stability test. Mechanistic investigations revealed that Na_2_WO_4_ promotes the dehydrogenation of diffused CH_4_ and CH_3_, leading to the formation of ^*^CH and ^*^CH_2_ intermediates. These species subsequently undergo C-C coupling on the Mn_3_O_4_ surface to form C_2_H_2_ and C_2_H_4_. The excellent catalytic performance, supported by in situ plasma-coupled FTIR characterization, is further corroborated by DFT calculations. These calculations demonstrate that a tandem catalytic effect is achieved through the isolated Na_2_WO_4_ and Mn_3_O_4_ active sites, which are responsible for the enhanced selectivity. Furthermore, the mesoporous nanoreactor design prevents the reduction of internal Mn_3_O_4_ by CH_4_ plasma through the Debye shielding effect. This reduces carbon deposition on the catalyst and protects the generated ^*^CH and ^*^CH_2_ intermediates from further decomposition due to the absence of plasma discharge within the mesopores. This catalyst design strategy offers significant potential for advancing plasma-catalysis. It enables highly selective and directional conversion, improving the energy efficiency of plasma-catalytic systems and paving the way for a high-value route to transform methane into unsaturated light olefins under mild conditions.

## Methods

### Synthesis of SiO_2_ nanospheres

Mesoporous SiO_2_ (m-SiO_2_) was synthesized using a double template method in an ethanol solution. First, ethanol and polyacrylic acid were added to a vial and stirred for 30 min. Then, diluted ammonia water was added to the above solution, followed by the introduction of polyether, and the mixture was stirred for another 30 min. After that, ethyl orthosilicate was added dropwise to the vial, resulting in a suspension after 4 h of stirring. Subsequently, the suspension was centrifuged and washed three times with ethanol. Finally, the precursors were evaporated overnight and calcination at 550 °C.

### Synthesis of α Na_2_WO_4_-β Mn_3_O_4_/m-SiO_2_

α Na_2_WO_4_/m-SiO_2_, β Mn_3_O_4_/m-SiO_2_, and α Na_2_WO_4_-β Mn_3_O_4_/m-SiO_2_ (where α and β denote the respective weight percentages of each metal oxide) were synthesized using the incipient wetness method. The Mn loading on the m-SiO_2_ support varied from 2 to 10 wt% (2, 5, 7.5, and 10 wt%), while the Na_2_WO_4_ loading ranged from 0.5 to 5% (0.5, 1, 2, and 5 wt%). For the preparation of Na_2_WO_4_/m-SiO_2_ and Mn_3_O_4_/m-SiO_2_, aqueous solutions of manganese nitrate tetrahydrate (Mn(NO_3_)_2_·4H_2_O) or sodium tungstate dihydrate (Na_2_WO_4_·2H_2_O) were mixed with m-SiO_2_ in a water bath. The mixture was stirred until it reached a paste-like consistency and then dried overnight at 70 °C. Subsequently, the dried samples were calcined in air at 550 °C. A two-step impregnation method was used for the synthesis of β Na_2_WO_4_-α Mn_3_O_4_/m-SiO_2_ (denoted as WMO/m-SiO_2_). First, Mn_3_O_4_/m-SiO_2_ was prepared as described above. Then, Na_2_WO_4_ was loaded onto the Mn_3_O_4_/m-SiO_2_ using the same drying and calcination procedures. This method was also used to prepare 1% Na_2_WO_4_-5% Mn_3_O_4_/SiO_2_ and 1% Na_2_WO_4_-5% Mn_3_O_4_/ZSM-5. Before testing, all catalysts were crushed and sieved to obtain particles with sizes between 30 and 60 mesh.

### Synthesis of Mn_3_O_4_-deposited m-SiO_2_ catalysts

Three types of catalysts with varying Mn_3_O_4_ locations were prepared: In-m-SiO_2_, where Mn_3_O_4_ was deposited exclusively within the mesopores of m-SiO_2_; Both-m-SiO_2_, where Mn_3_O_4_ was partially distributed within the mesopores and on the exterior surface of m-SiO_2_; Out-m-SiO_2_, where Mn_3_O_4_ was primarily located on the exterior surface of m-SiO_2_. In-m-SiO_2_ was synthesized as described above. Both-m-SiO_2_ was prepared using a rapid heating and drying method, where Na_2_WO_4_ was first loaded onto m-SiO_2_, followed by the deposition of Mn_3_O_4_. The precursors were rapidly heated from room temperature to 300 °C at a heating rate of 20 °C min^−1^ for 2 h and then calcined at 550 °C. Out-m-SiO_2_ was synthesized by mechanically mixing pre-synthesized Mn-O and Na-W-O precursors. Specifically, solutions of Mn or W were dissolved in deionized water and stirred at 50 °C for 3 h. After drying overnight, the precursors were mechanically mixed with m-SiO_2_ and calcined at 550 °C.

### Catalyst activity test

The performance of the catalyst in the thermal catalytic NOCM reaction (denoted as catalyst only) was evaluated in a DBD reactor (plasma off) equipped with heating tape and operated at atmospheric pressure. The reactor was loaded with 1% Na_2_WO_4_-5% Mn_3_O_4_/m-SiO_2_ and secured with quartz wool on both ends. Prior to testing, the catalyst was pretreated with argon (200 mL min^−1^) at 100 °C for 20 min to remove impurities. The temperature was then increased to 250 °C and monitored using a K-type thermocouple placed within the catalyst bed. A diluted methane feed (5 vol% CH_4_ in Ar) at a total flow rate of 200 mL min^−1^ was used to minimize mass transfer limitations and accurately evaluate the intrinsic catalytic activity. The feed gas was preheated to 30 °C, and the experiments were conducted for 60 min.

For plasma-catalysis and plasma-only conditions, the DBD reactor was operated without external heating. The experimental procedure involved the following steps: (1) The catalyst was pretreated with argon (100 mL min^−1^) at 100 °C for 20 min, followed by cooling to room temperature. (2) A 5 vol% CH_4_/Ar mixture (200 mL min^−1^) flowed through the DBD reactor for 10 min before switching on the plasma. (3) The plasma was ignited at an SEI of 5.1 kJ L^−1^ (calculated as the discharge power divided by the gas flow rate) and maintained at a discharge power of 17 W with a flow rate of 200 mL min^−1^ for 60 min. (4) After switching off the plasma, the DBD reactor was purged with argon (100 mL min^−1^) for 10 min. (5) The spent catalyst was removed, and a 10 vol% O_2_/Ar mixture (100 mL min^−1^) was introduced to oxidize solid carbon deposited in the DBD reactor. This step was carried out at an SEI of 6.14 kJ L^−1^ until no CO or CO_2_ was detected in the exhaust gas. (6) The spent catalyst was reintroduced into the cleaned reactor, and a 10 vol% O_2_/Ar mixture (100 mL min^−1^) was used to oxidize carbon deposited on the catalyst. This step was continued, and no CO or CO_2_ were detected in the exhaust gas. The amount of carbon deposited was calculated using the equation *N*_C_ = *V* × (*C*_CO2_ + *C*_CO_) × 22.4, where *V* is the total volume of exhaust gas during the oxidation process, and *C*_CO2_ and *C*_CO_ are the concentrations of CO_2_ and CO in the exhaust gas, respectively. To avoid interfering with the plasma field, the reactor temperature was measured using an infrared thermometer.

### Isotopic labeling experiments

To investigate the relative contributions of CH_2_ and CH_3_ radicals to CH_4_ decomposition, isotopic labeling experiments were conducted. The catalyst was first pretreated in a 5 vol% CH_4_/Ar mixture (discharge power 17 W, flow rate 200 mL min^−1^) for 60 min and then cooled to room temperature. Next, argon (200 mL min^−1^) was flowed through the reactor for 10 min to purge residual gases. A gas mixture of 2.5 vol% CD_4_ and 2.5 vol% CH_4_ in argon (200 mL min^−1^) was subsequently introduced into the DBD reactor for 10 min. The plasma was then switched on and sustained for 60 min, during which mass spectrometric signals at m/z = 15, 18, and 19 were monitored. The concentrations of CH_4_ and CD_4_ in the mixture were determined using mass spectrometry. From these measurements, the conversion of CH_4_ and CD_4_, as well as the selectivity of the product, was determined. Under plasma discharge conditions, CH_4_ and CD_4_ dissociate into radicals including CH_3_, CH_2_, CH, CD_3_, CD_2_, CD, H, and D. These radicals recombine to form isotopically labeled methane species, such as CD_3_H (m/z = 19), and CD_2_H_2_ (m/z = 18). The formation of CD_3_H and CD_2_H_2_ results from the recombination reactions of CD_3_ with H and CD_2_ with two H atoms, respectively. The signals at m/z = 15 and m/z = 19 reflect the concentrations of CH_4_ and CD_3_H, respectively. When analyzing the peak intensity of CD_2_H_2_, it is critical to consider the contribution of CD_4_ fragmentation (m/z = 20), which generates CD_3_ (m/z = 18). Specifically, the signal at m/z = 18 reflects contributions from both CD_2_H_2_ and the CD_3_ fragment derived from CD_4_ fragmentation.

### In situ plasma-coupled FTIR characterization

To elucidate plasma-induced surface reactions during the plasma-catalytic NOCM process, we employed in situ plasma-coupled FTIR spectroscopy using a custom-designed plasma gas cell^19^. The experimental procedure is described in detail as follows: (I) Prior to analysis, each sample was pretreated with argon plasma (99.999% purity) at a flow rate of 100 mL min^−1^ in a DBD reactor at 100 °C for 20 min to remove residual surface species. (II) A 5% CH_4_/Ar mixture (40 mL min^−1^) was introduced to purge the cell for 30 min. During this step, the temperature was decreased from 100 to 35 °C, after which the IR background spectrum was collected. (III) The plasma was switched on, and the plasma-catalytic NOCM was conducted for 15 min. (IV) After switching off the gas flow, IR spectra were collected every 3 min for a total duration of 18 min.

Quasi-in situ diffuse reflectance infrared Fourier transform spectroscopy (DRIFT) analysis was conducted using an FTIR spectrometer (IS50, Thermo Fisher Co. Ltd.) equipped with a liquid nitrogen N_2_-cooled mercury-cadmium-telluride (MCT) detector. The background spectrum was obtained by pretreating the catalyst with Ar plasma in a DBD reactor under conditions identical to those used in the plasma-catalytic NOCM experiments (SEI = 5.1 kJ L^−1^, flow rate = 200 mL min^−1^). Following pretreatment, the catalyst was cooled to room temperature before conducting the quasi-in situ DRIFTS measurements. To avoid air exposure, the pretreated catalysts were transferred from the DBD reactor to the DRIFT cell within a glovebox. For the plasma-catalytic NOCM reaction, the plasma was switched off after the experiment, and the inlet and outlet of the DBD reactor were sealed. Subsequently, the spent catalysts were then transferred from the DBD reactor to the DRIFT cell within a glovebox, and IR spectra were collected at room temperature.

### Computational details

Spin-polarized density functional theory (DFT)^39,40^ calculations were performed using the Vienna ab initio simulation package (VASP) code^41^. The exchange-correlation interactions between electrons were described using the Perdew–Burke–Ernzerhof (PBE) functional within the generalized gradient approximation (GGA)^42,43^. Following convergence tests, plane-wave pseudopotentials with kinetic cutoff energy of 420 eV^44^ for Mn_3_O_4_ and 500 eV^45^ for Na_2_WO_4_ method were employed within the projector augmented wave (PAW) method. The Mn_3_O_4_ (211) and Na_2_WO_4_ (111) surfaces were selected as the computational models based on the experimental results (Fig. 1e and Supplementary Fig. 3). To minimize interactions between the slab and its periodic images, a vacuum layer of ~15 Å was added above the slab. During geometry optimization, the bottom two atomic layers were fixed, while all other atoms and adsorbates were allowed to relax until the force on each atom was less than 0.01 eV Å^−1^. A convergence criterion of 1 × 10^−5^ eV/atom was used for structural optimization. Brillouin zone integration was performed using a 2 × 2 × 1 Monkhorst-Pack grid with a Methfessel-Paxton smearing width (σ) of 0.2 eV. Due to the presence of localized 3*d* states on Mn, the electronic structure of Mn was treated within the DFT + U formalism with a U-J parameter of 4.00 eV^46^. In addition, to account for weak interactions within the catalyst, van der Waals corrections were incorporated using the DFT-PBE-D3 method^47^. Further details regarding the DFT calculation methods are provided in the Supplemental Information.

## Supplementary information

Supplementary Information

Transparent Peer Review File

## Source data

Source Data

## Data Availability

The data presented in the figures and the key findings of this study are available from the corresponding authors upon reasonable request. Source data are provided with this paper.
