# Supplementary material for: Shielded bifunctional nanoreactor enabled tandem catalysis for plasma methane coupling
Source: Nat Commun. 2025 May 17;16:4585. doi: 10.1038/s41467-025-59709-y (PMC12085660; doi:10.1038/s41467-025-59709-y)
Supplement: Supplementary file 1 — Supplementary Information [file 41467_2025_59709_MOESM1_ESM.pdf]

Supplementary Information

Shielded Bifunctional Nanoreactor Enabled Tandem Catalysis for Plasma Methane Coupling

Chunqiang Lu[1, 2#], Yaolin Wang[3#], Dong Tian[1, 2#], Ruidong Xu[1, 2], Roong Jien Wong [4, 5], Shibo Xi[5], Wen Liu[4], Hua Wang[1, 2, *], Xin Tu[3, *], Kongzhai Li[1, 2, 6, *]

[1] State Key Laboratory of Complex Nonferrous Metal Resources Clean Utilization, Kunming University of Science and Technology, Kunming 650093, P. R. China

[2] Faculty of Metallurgical and Energy Engineering, Kunming University of Science and Technology, Kunming 650093, P. R. China

[3] Department of Electrical Engineering and Electronics, University of Liverpool, Liverpool L69 3GJ, UK

[4] School of Chemistry, Chemical Engineering and Biotechnology, Nanyang Technological University, 62 Nanyang Drive, Singapore 637459, Singapore

[5] Institute of Sustainability for Chemicals, Energy and Environment (ISCE2), Agency for Science, Technology and Research (A*STAR), 1 Pesek Road, Jurong Island, Singapore 627833, Republic of Singapore.

[6] Southwest United Graduate School, Kunming 650092, P. R. China

[#]*These authors contributed equally to this work.*

*Corresponding authors: xin.tu@liv.ac.uk (X.T.); kongzhai.li@foxmail.com (K.L.); wanghua65@163.com (H.W.)

1 Supplementary method

1.1 Chemicals

$Na_2WO_4 \cdot 2H_2O$, $Mn(NO_3)_2 \cdot 4H_2O$, ethanol, polyacrylic acid (PAA), ammonia, polyether (P123), ethyl orthosilicate (EOS) cetyltrimethylammonium bromide (CTAB), SiO_2 and a molecular sieve (ZSM-5) were purchased from commercial sources (Aladdin and Alfa Aesar) without undergoing any further purification. The experiments used argon (Ar), diluted methane (5 vol% CH_4/Ar), diluted oxygen (10 vol% O_2/Ar) and N_2, all supplied by Messer Co., Ltd.

1.2 Catalyst characterization

X-ray diffraction (XRD) analysis was performed on a MiniFlex600 diffractometer (Rigaku) using Cu Kα radiation (λ = 0.15406 nm) to investigate the crystalline structure of all samples. Data were collected over a 2θ range of 10° and 90° at a scan rate of 2° min^{-1}. The surface morphology and microstructure of the samples were examined using a scanning electron microscope (SEM) (VERSA 3D, FEI) and a field-emission transmission electron microscope (TEM) (FEI TECNAI G2 F20 300 kV) equipped with an energy-dispersive X-ray (EDX) analyzer. The Brunauer–Emmett–Teller (BET) surface area was measured via nitrogen adsorption at 77 K using a Quantachrome AutoSorb-iQ instrument.

The surface elemental states of the catalysts were characterized by quasi-*in situ* X-ray photoelectron spectroscopy (XPS) using an ESCALAB250 spectrometer (Thermo VG, USA) equipped with an Al Kα (1486.6 eV) X-ray source. All binding energies

were calibrated using the C 1*s* peak at 284.80 eV. Prior to XPS measurements, the reference catalysts were placed on a sample holder in a glovebox to avoid air exposure. X-ray absorption near edge structure (XANES) and extended X-ray absorption fine structure (EXAFS) data were collected at the XAFCA beamline of the Singapore Light Source, Singapore Synchrotron Radiation Facility (SSRF).

1.3 Plasma reactor and electrical measurements

The reaction was conducted in a dielectric barrier discharge (DBD) plasma reactor with a discharge length of 50 mm and a discharge gap of 2 mm (see Supplementary Fig. 1). A stainless steel mesh wrapped around a quartz tube (with an inner diameter of 8 mm and a wall thickness of 2 mm) served as the high-voltage electrode. A corundum tube (inner diameter: 2 mm, wall thickness: 1 mm), positioned along the axis of the quartz tube, housed a 2-mm diameter stainless-steel rod that served as the ground electrode.

A high-voltage AC power source (CTP-2000K, Nanjing Suman Electronic Co. Ltd) was used to power the DBD reactor. The applied voltage of the DBD was measured by a high-voltage probe (Tektronix, P6015A), while the current was recorded by measuring the voltage drop across an integrated resistor (50 Ω) using a voltage probe. An external capacitor (0.47 μF) was used to measure the charge formed during the discharge. All electrical signals were recorded by a digital oscilloscope (SIGLENT, SDS1102X-C). The discharge power was calculated using the typical Q-U Lissajous figure method[1].

1.4 Methane coupling experiments

During the plasma catalytic non-oxidative coupling of methane (NOCM) reaction, gas samples were collected at regular intervals (0-2 min, 2-4 min, 4-6 min, 6-8 min, 8-10 min, 10-15 min, 15-20 min, 20-25 min, 25-30 min, 30-40 min, 40-50 min, and 50-60 min). These samples were analyzed using a gas chromatograph (GC 2060, Shanghai Ruimin Co., Ltd.) equipped with a flame ionization detector (FID) and a thermal conductivity detector (TCD). The outlet flow was continuously monitored online using a mass spectrometer (AMTEK Dycor System 200 LCD, Pfeiffer Vacuum).

In the plasma-driven NOCM reaction, methane is converted into C_2-C_3 hydrocarbons (C_2H_2, C_2H_4, C_2H_6, and C_3H_8), hydrogen, and solid carbon. The following equations were used to calculate the key reaction performance metrics, including CH_4 conversion (X_{CH4}), hydrocarbon selectivity (R), solid carbon selectivity on the catalyst (R_{carbon}), molar fraction of hydrocarbons (F), yields of C_2H_2 and C_2H_4 (Y), equivalent carbon deposition rate (ECR), energy cost for CH_4 conversion (EC_{CH4}) and specific energy input (SEI). The molar fraction of a specific hydrocarbon (C_2H_2, C_2H_4, C_2H_6, or C_3H_8) or a mixture of C_2H_2 and C_2H_4 is defined as the percentage of the molar amount of carbon in that specific hydrocarbon (or mixture) relative to the total molar amount of carbon in all C_2-C_3 hydrocarbons (Eq. 4). The equivalent carbon deposition rate is defined as the solid carbon selectivity on the catalyst (R_{carbon}) relative to CH_4 conversion (X_{CH4}):

$$X_{CH4}\ (\%) = \frac{N_{in} - N_{out}}{N_{in}} \times 100\% \quad (1)$$

$$R\ (\%) = \frac{N_{\text{product}}}{N_{\text{in}} - N_{\text{out}}} \times 100\% \tag{2}$$

$$R_{carbon}\ (\%) = \frac{N_{\text{carbon}}}{N_{\text{in}} - N_{\text{out}}} \times 100\% \tag{3}$$

$$F\ (\%) = \frac{N_{\text{product}}}{N_{\text{total}}} \times 100\% \tag{4}$$

$$Y\ (\%) = X_{\text{CH4}} \times R_{\text{C2H2/C2H4}} \tag{5}$$

$$\text{ECR} = \frac{R_{\text{carbon}}}{X_{\text{CH4}}} \tag{6}$$

$$\text{EC}_{\text{CH4}}\ (\text{MJ/mol}) = \frac{W_{\text{discharge}}}{N_{\text{CH4}}} \tag{7}$$

$$\text{SEI}\ (\text{kJ/L}) = \frac{W_{\text{discharge}}}{V} \tag{8}$$

Where N_{in} and N_{out} are the molar amount of CH_4 in the inlet and outlet gases, respectively. N_{carbon} is the molar amount of carbon deposited on the catalyst. $N_{product}$ represents the molar amount of carbon in each hydrocarbon product (C_2H_2, C_2H_4, C_2H_6, or C_3H_8), N_{total} is the total molar amount of carbon in all hydrocarbons (C_2H_2, C_2H_4, C_2H_6, and C_3H_8) after the plasma reaction reaches a stable state. $W_{discharge}$ is the discharge power, and N_{CH4} represents the molar amount of converted CH_4. V is the total flow rate of inlet gas.

The carbon balance was calculated as the total molar amount of carbon in all measured carbon-containing products (C_2H_2, C_2H_4, C_2H_6, C_3H_8, and deposited carbon in the reactor and on the catalyst) divided by the total molar amount of carbon in the inlet methane stream. Error bar is determined by three independent replicate experiments.

1.5 DFT calculations

Spin-polarized density functional theory (DFT)[2,3] calculations were performed using the Vienna ab initio simulation package (VASP, version 6.21) code[4]. The exchange-correlation interactions between electrons were described using the Perdew–Burke–Ernzerhof (PBE) functional within the generalized gradient approximation (GGA)[5,6]. Plane wave pseudopotentials with kinetic energy cutoffs of 420 eV[7] for Mn_3O_4 and 500 eV[8] for Na_2WO_4 were employed within the projector augmented wave (PAW) method, as determined by convergence tests. The Mn_3O_4 (211) and Na_2WO_4 (111) surfaces were selected as computational models based on experimental results (Fig. 1e and Supplementary Fig. 3). The DFT-calculated lattice constants for bulk Mn_3O_4 were a = b = 5.841 Å and c = 9.462 Å, while for Na_2WO_4, a = b = c = 9.157 Å. These values were all in excellent agreement with previously reported values, a = b = 5.840 Å and c = 9.500 Å for Mn_3O_4[9], and a = b = c = 9.129 Å for Na_2WO_4[9,10]. The Mn_3O_4 (211) (Supplementary Fig. 35 (a, b)) and Na_2WO_4 (111) (Supplementary Fig. 35 (c, d)) surfaces were modeled using a four-layer 2×2 surface slab. A vacuum layer with a thickness of ~15 Å was added to the slab to eliminate unphysical interactions between periodic images perpendicular to the surface. During geometry optimization, atoms in the bottom two layers were fixed, while all other atoms, including adsorbates, were allowed to relax until the force on each ion was less than 0.01 eV $Å^{-1}$. The convergence criterion for structure optimization was set to 1×10^{-5}. Brillouin-zone integration was performed using a 2×2×1 Monkhorst-Pack grid with Methfessel-Paxton smearing (σ = 0.2 eV). To account for on-site coulomb interactions, the

electronic structure of Mn was treated using the DFT+U formalism, with a parameter U-J = 4.00 eV applied to the localized 3d states of Mn[11]. In addition, van der Waals corrections were incorporated using the DFT-PBE-D3 method to accurately describe weak interactions with the catalyst[12].

As shown in Supplementary Fig. 35(a-d), Supplementary Tables 4 and 5, different potential adsorption sites of intermediates for the plasma-catalytic NOCM to C_2H_2 and C_2H_4 on Mn_3O_4 (211) and Na_2WO_4 (111) surfaces were considered during the calculation of binding energies (*BE*). The *BE* of each adsorbate was calculated as follow[12-15]:

$$BE_{\text{adsorbate}} = E_{\text{slab+adsorbate}} - E_{\text{adsorbate}} - E_{\text{slab}} \quad (9)$$

where $E_{\text{slab+adsorbate}}$, $E_{\text{adsorbate}}$, and E_{slab} represent the total energies of the slab with adsorbate, the adsorbate species in the gas phase, and the clean slab, respectively. To further investigate the influence of SiO_2 on the adsorption of intermediates during the plasma-catalytic NOCM to C_2H_2 and C_2H_4, we constructed $NaWO_4/SiO_2$ and Mn_3O_4/SiO_2 models using an inverse modeling approach. Specifically, a small SiO_2 cluster (Si_8O_4) was deposited on four-layer 2×2 Mn_3O_4 (211) and Na_2WO_4 (111) surfaces (Supplementary Fig. 36). The potential adsorption sites of intermediates on SiO_2/Mn_3O_4 (211) and SiO_2/Na_2WO_4 (111) were also analyzed to elucidate the influence of SiO_2 (Supplementary Fig. 36, 39, 40 and 41; Supplementary Tables 6 and 7).

The Gibbs free energy (*G*) of a species was calculated using the following equation[14]

$$G = E + \text{ZPE} - T \times S \quad (10)$$

where E is the total energy of a species obtained from DFT calculations, ZPE is the zero-point energy, S is the entropy, and T is the temperature (523.15 K in this study). Vibrational modes were determined using DFT calculations with reasonable accuracy. The entropy of a species was calculated using the harmonic normal mode approximation based on DFT-calculated vibrational frequencies[15]:

$$S_{vib}=k_B \sum_{i}^{\#\ of\ modes}\left(\frac{\chi_i}{e^{\chi_{i-1}}}-\ln(1-e^{-\chi_i})\right) \qquad (11)$$

where χ_i for each vibrational mode is defined based on the vibrational frequency, v_i, as described[16]

$$\chi_i=\frac{\hbar v_i}{k_B T} \qquad (12)$$

The ZPE was calculated from v_i as follows[14:]

$$E_{ZPE}=\sum_{i=1}^{number\ of\ modes}\frac{1}{2}h v_i \qquad (13)$$

Here, k_B and $\hbar$ are the Boltzmann constant and Planck's constant, respectively, and T is the temperature. Thus, the ZPE and entropy contributions were determined using DFT calculations.

The change in free energy (ΔG) was calculated using the following equation[14-16]:

$$\Delta G = \Delta E + \Delta ZPE - T\times\Delta S \qquad (14)$$

Where ΔE is the binding energy of the adsorbed species.

The climbing image nudged elastic band (CI-NEB) method, implemented in VASP, was used in conjunction with the built-in Dimer method to identify transition states of chemical reactions. This approach has proven highly effective for determining activation energies. The activation energy (E_a) of a chemical reaction is defined as the energy difference between the transition state (TS) and the initial state (IS), while the

reaction energy (ΔE) is the energy difference between the final state (FS) and the initial state. The activation energy and the reaction energy were calculated using the following equations[12,16].

$$E_a = E_{TS} - E_{IS} \quad (15)$$

$$\Delta E = E_{FS} - E_{IS} \quad (16)$$

where E_{IS}, E_{TS} and E_{FS} are the total energies of the initial state, transition state and final state, respectively.

2 Supplementary Figures

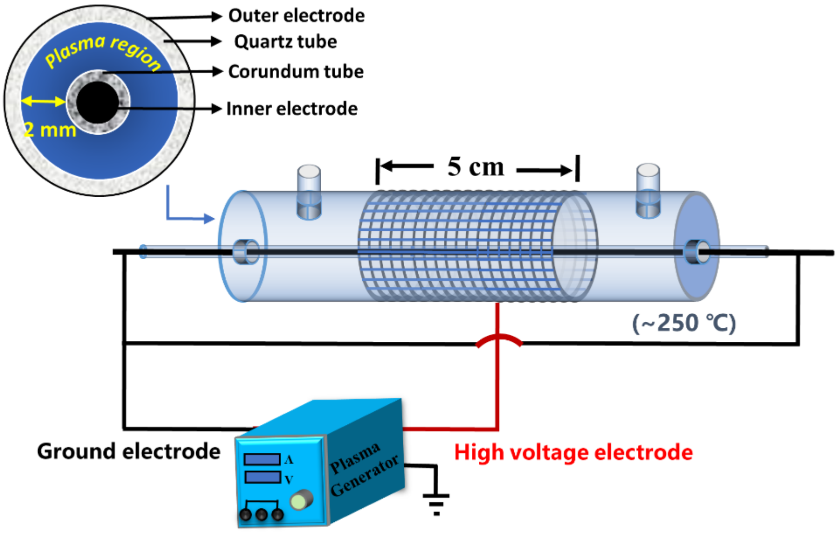

Supplementary Fig. 1 Schematic diagram of the DBD reactor.

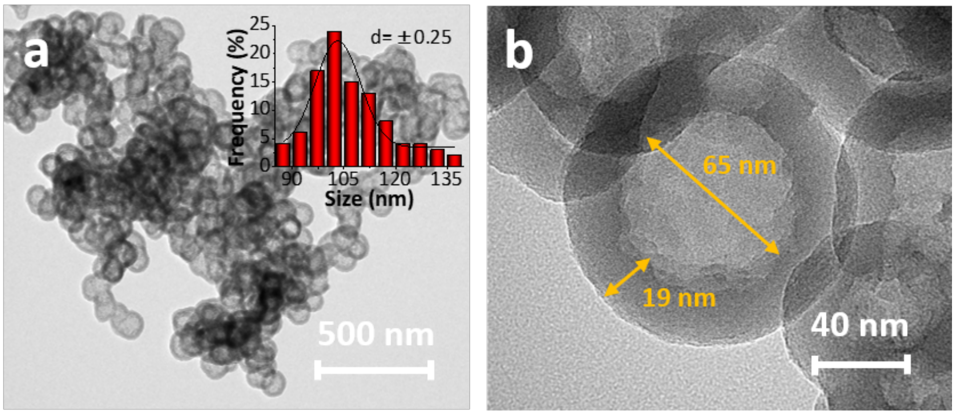

Supplementary Fig. 2 TEM images of m-SiO_2 nanospheres (a and b).

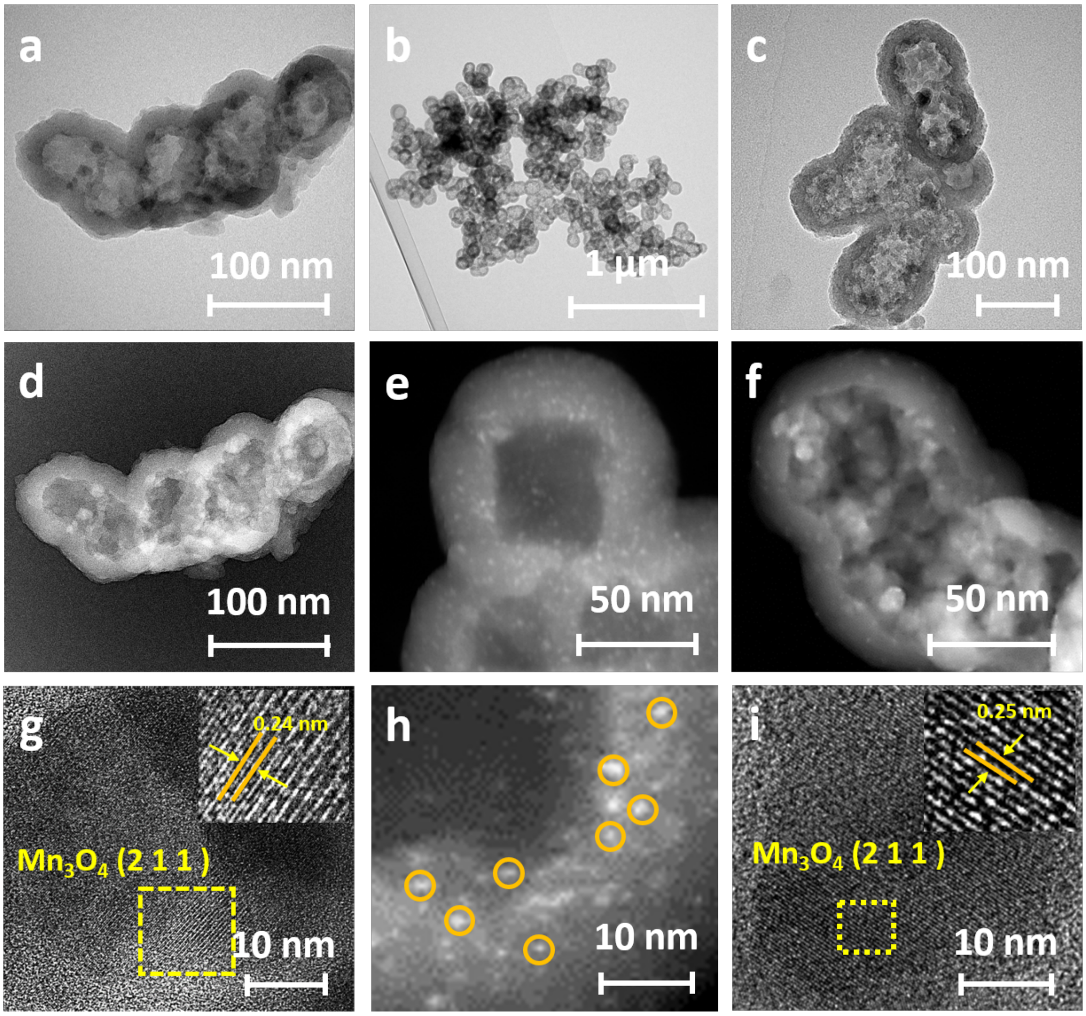

Supplementary Fig. 3 TEM, STEM and HRTEM images of (a, d and g) 5% Mn_3O_4/m-SiO_2, (b, e and h) 1% Na_2WO_4/m-SiO_2 and (c, f and i) WMO/m-SiO_2. Here, isolated tungstate species particles (1.5-2 nm) are immobilized in the m-SiO_2 shell.

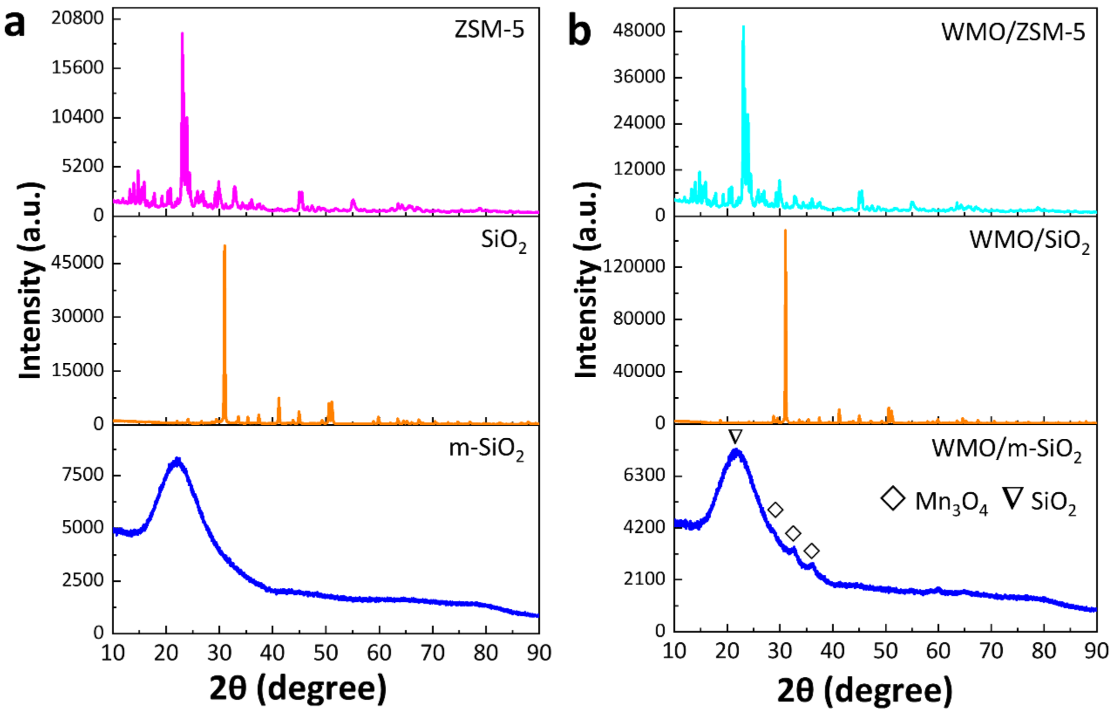

Supplementary Fig. 4 XRD patterns of different samples. (a) ZSM-5, SiO_2 and m-SiO_2. (b) WMO/ZSM-5, WMO/SiO_2 and WMO/m-SiO_2.

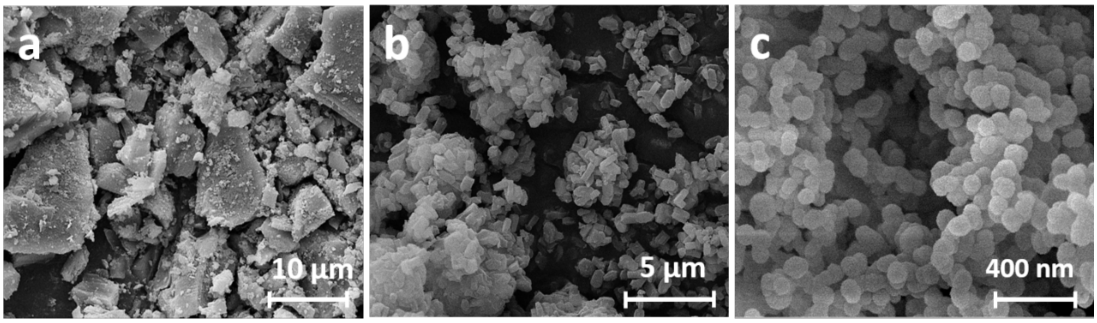

Supplementary Fig. 5 SEM images of different catalysts. (a) WMO/SiO_2. (b) WMO/ZSM-5. (c) WMO/m-SiO_2.

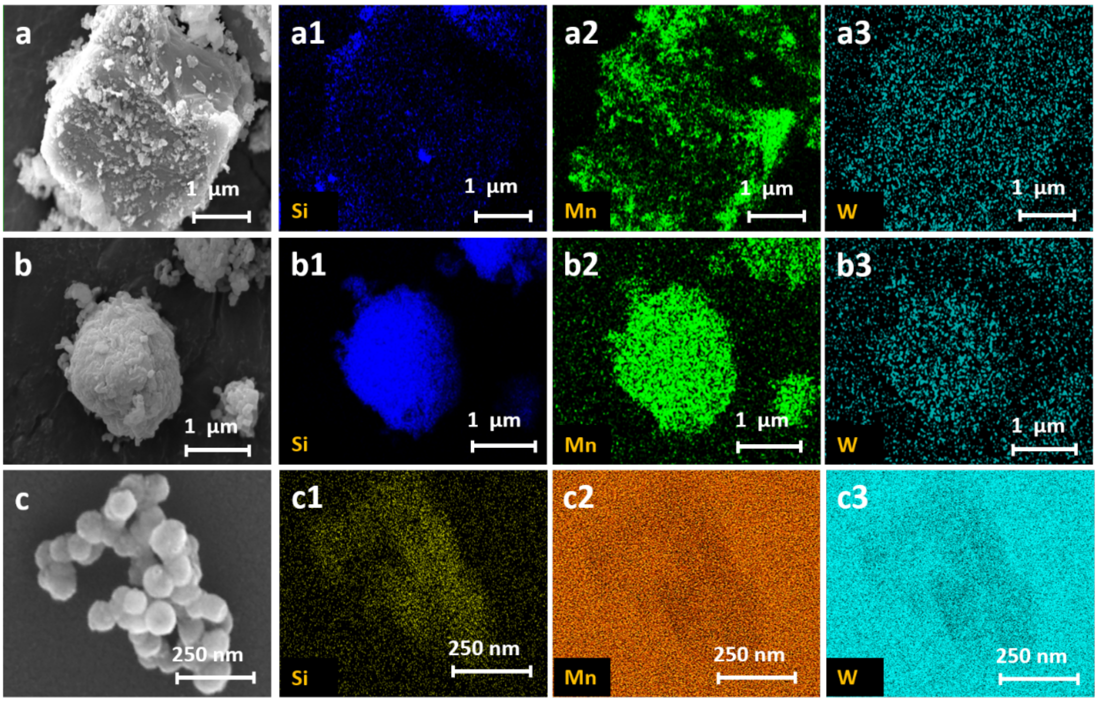

Supplementary Fig. 6 Elemental distribution maps of (a-a3) WMO/SiO_2. (b-b3) WMO/ZSM-5. (c-c3) WMO/m-SiO_2. W and Mn were not detected on the surface (c2 and c3), indicating that W and Mn species were possibly encapsulated by m-SiO_2.

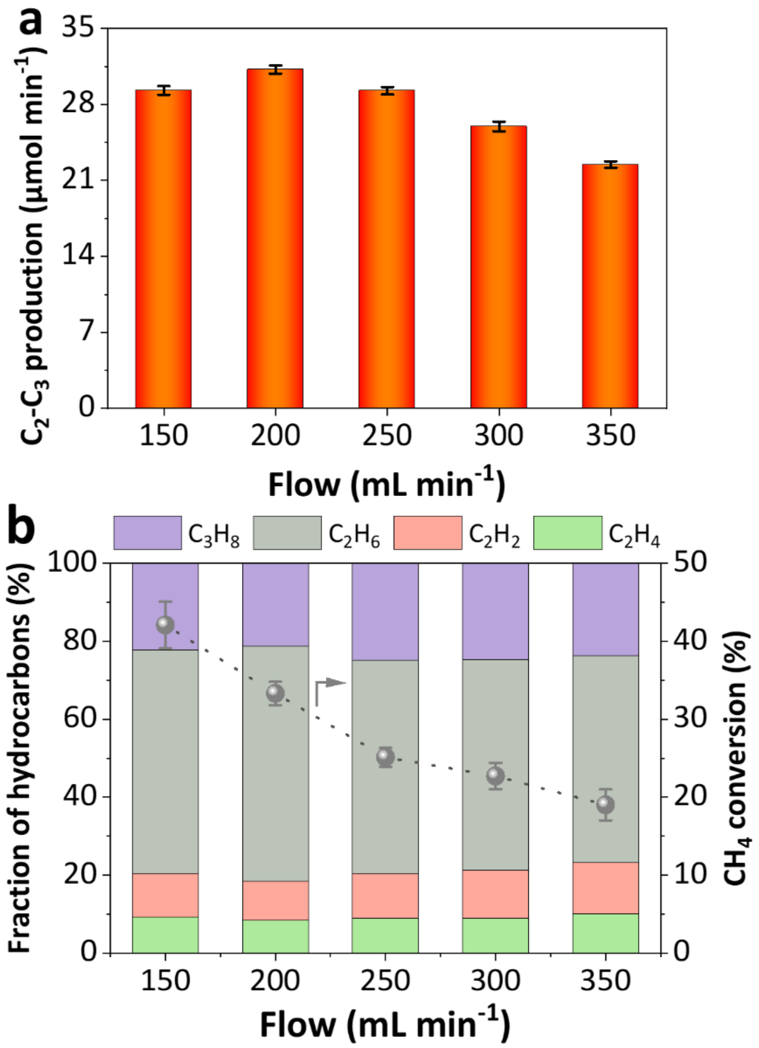

Supplementary Fig. 7 (a) Production of C_2-C_3 hydrocarbons (C_2H_2 and C_2H_4, C_2H_6 and C_3H_8) under plasma-only conditions at various flow rates. (b) Molar fraction of hydrocarbons and CH_4 conversion under plasma-only conditions at various flow rates (Conditions: 1 bar, discharge power 17 W, experiment duration 60 min). Error bars (standard deviation) in the figure were obtained from three sampling runs.

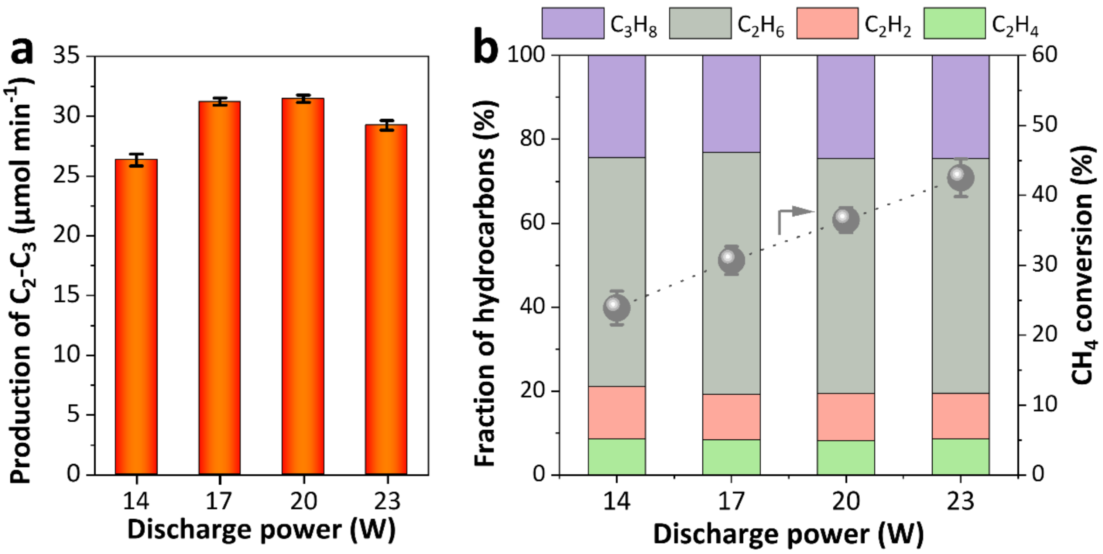

Supplementary Fig. 8 (a) Effect of discharge power on the production of C_2-C_3 hydrocarbons (C_2H_2, C_2H_4, C_2H_6 and C_3H_8). (b) Effect of discharge power on molar fraction of hydrocarbons and CH_4 conversion (Conditions: Feed gas 5 vol% CH_4/Ar, total flow rate 200 mL min^{-1}, discharge powers 14, 17, 20, and 23 W). Error bars (standard deviation) in the figure were obtained from three sampling runs.

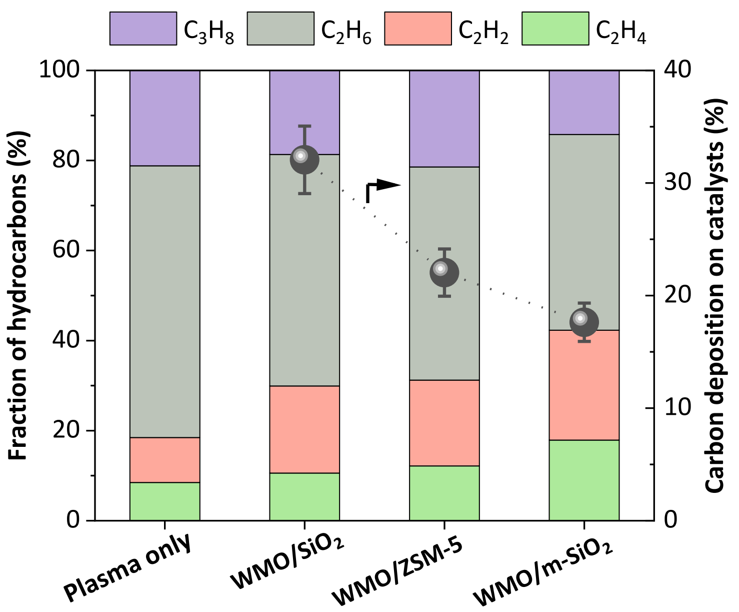

Supplementary Fig. 9 Effect of catalysts on CH_4 conversion and carbon deposition on catalysts (Conditions: 1 bar, SEI 5.1 kJ L^{-1}, total flow rate 200 mL min^{-1}, discharge power 17 W, experiment duration 60 min). Error bars (standard deviation) in the figure were obtained from three sampling runs.

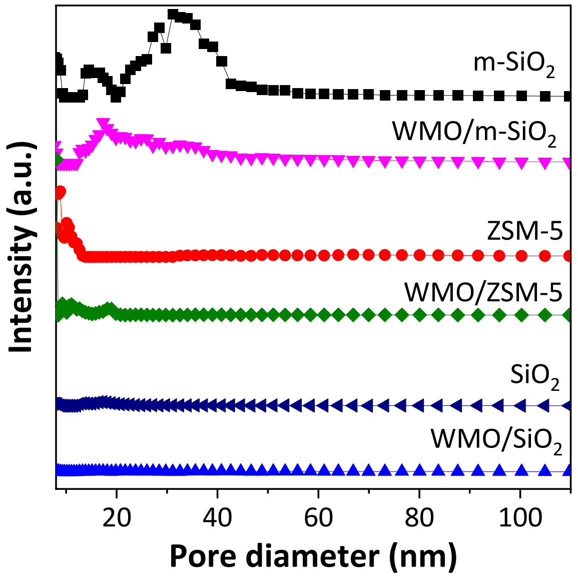

Supplementary Fig. 10 Pore size distributions of the catalysts.

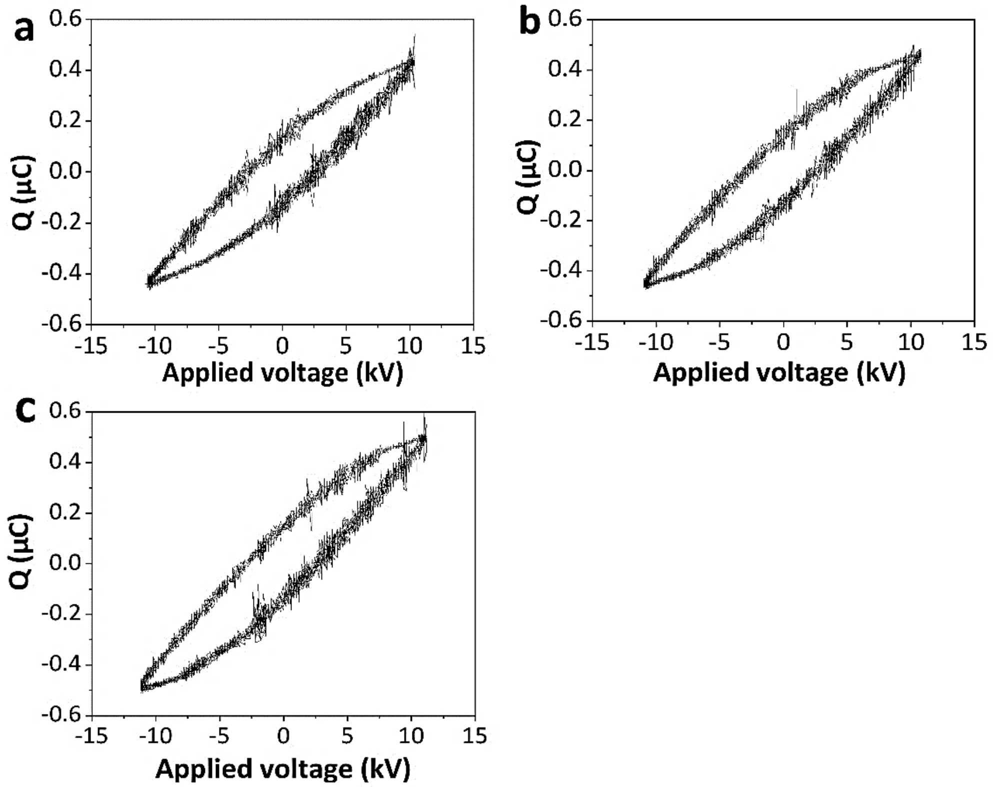

Supplementary Fig. 11 Lissajous figures. (A) SiO_2. (B) ZMS-5. (C) m-SiO_2 (Conditions: 1 bar, SEI 5.1 kJ L^{-1}, total flow rate 200 mL min^{-1}, discharge power 17 W, experiment duration 60 min).

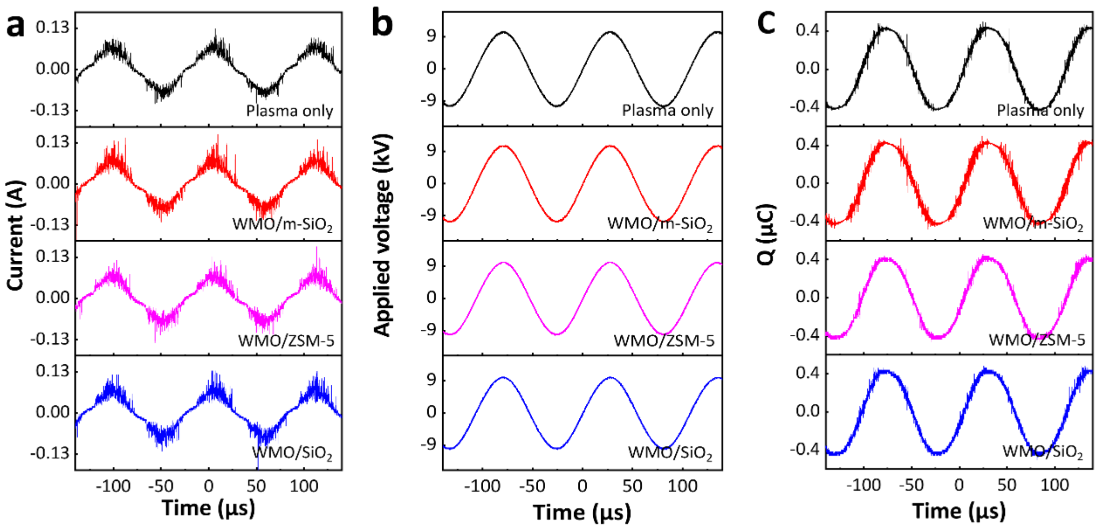

Supplementary Fig. 12 Electrical signals for plasma-only and plasma with different catalysts (WMO/m-SiO_2, WMO/ZSM-5 and WMO/SiO_2). (a) Current. (b) Applied voltage. (c) Charge (Conditions: 1 bar, SEI 5.1 kJ L^{-1}, total flow rate 200 mL min^{-1}, discharge power 17 W, experiment duration 60 min).

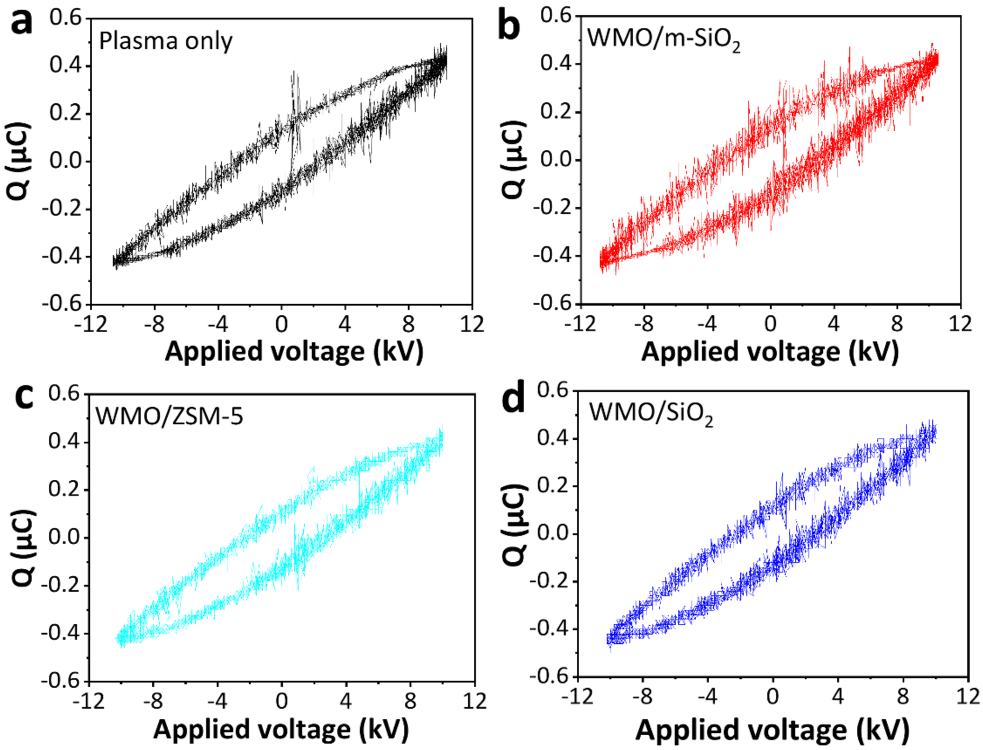

Supplementary Fig. 13 Lissajous figures. (a) Plasma only. (b) Plasma with WMO/m-SiO_2. © Plasma with WMO/ZSM-5. (d) Plasma with WMO/SiO_2 (Conditions: 1 bar, SEI 5.1 kJ L^{-1}, total flow rate 200 mL min^{-1}, discharge power 17 W).

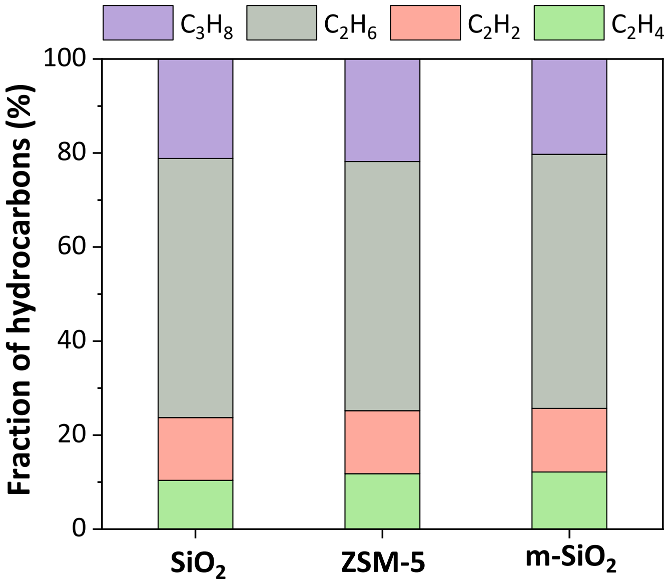

Supplementary Fig. 14 Molar fractions of C_2-C_3 hydrocarbons using SiO_2, ZSM-5 and m-SiO_2 (Conditions: 1 bar, SEI 5.1 kJ L^{-1}, total flow rate 200 mL min^{-1}, discharge power 17 W, experiment duration 60 min).

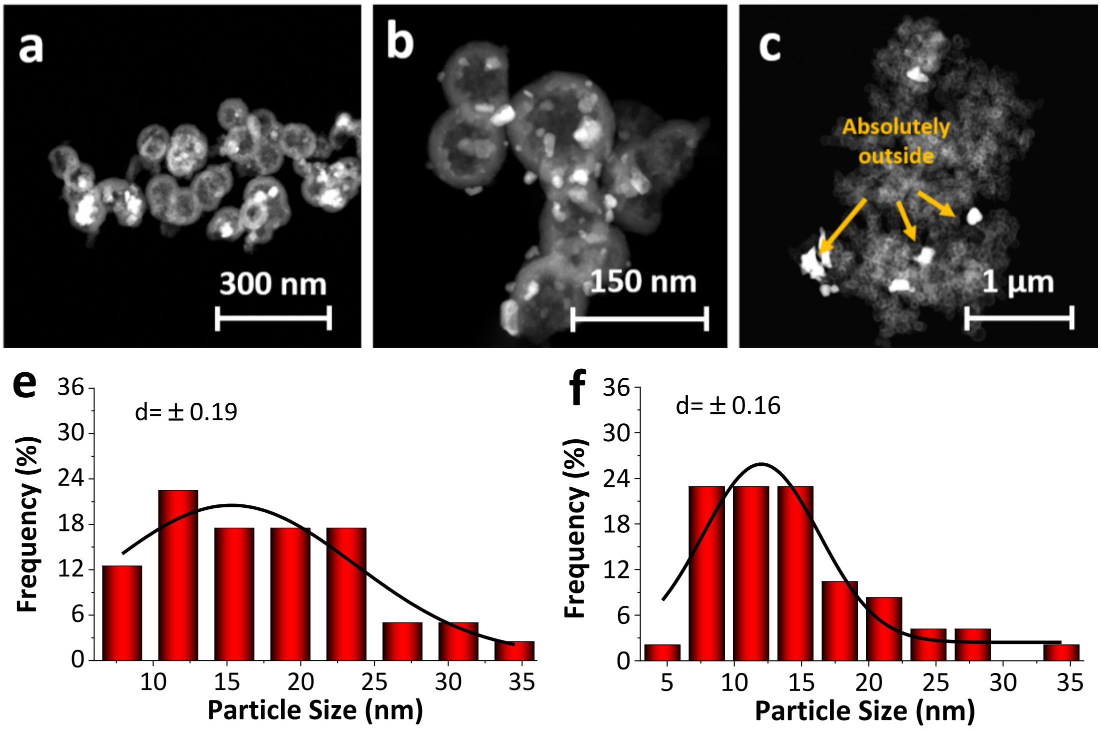

Supplementary Fig. 15 Scanning transmission electron microscope (STEM) images of (a) In-m-SiO_2. (b) Out-m-SiO_2. (c) Both-m-SiO_2. Mn_3O_4 particle size distributions of (e) In-m-SiO_2 and (f) Out-m-SiO_2. The catalyst particles are exclusively deposited inside m-SiO_2 (In-m-SiO_2), are partially distributed within m-SiO_2 (Both-m-SiO_2) and are predominantly deposited on the outside of m-SiO_2 (Out-m-SiO_2).

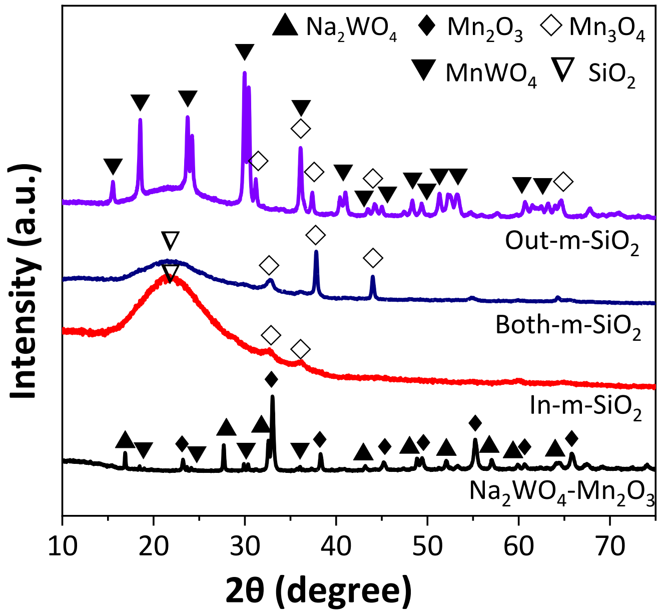

Supplementary Fig. 16 XRD patterns of the catalysts.

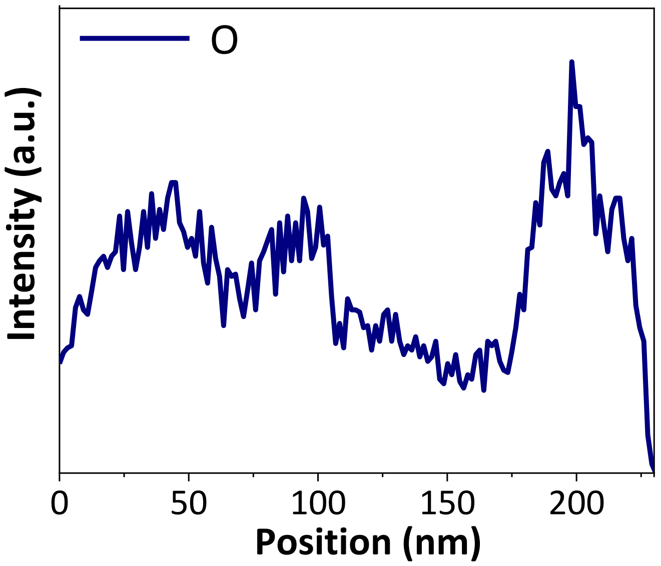

Supplementary Fig. 17 TEM-EDS line scans of oxygen on Both-m-SiO_2 (based on Fig. 2a).

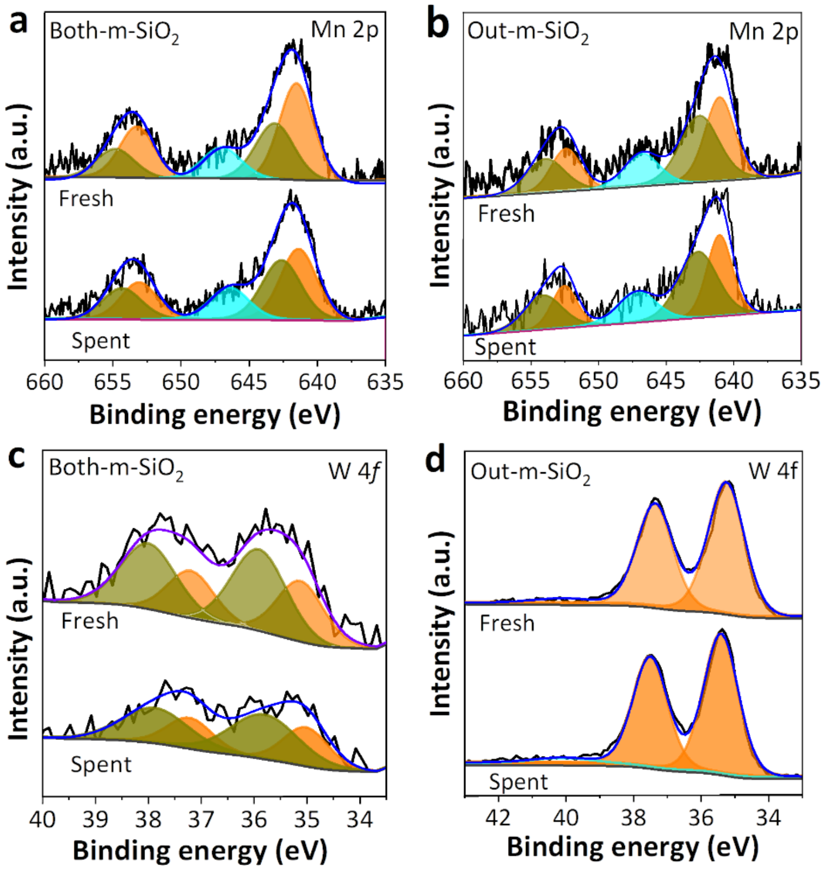

Supplementary Fig. 18 XPS spectra in the Mn 2*p* and W 4*f* regions (from top to bottom) of fresh Both-m-SiO_2 and Out-m-SiO_2, as well as spent Both-m-SiO_2 and Out-m-SiO_2.

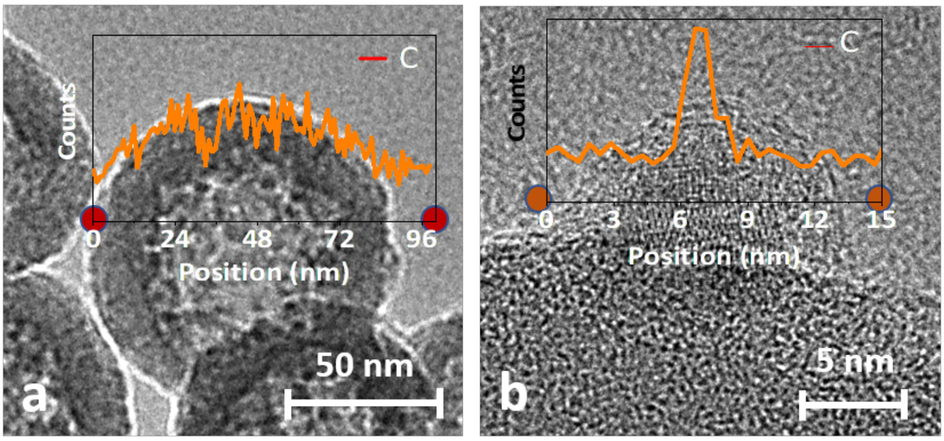

Supplementary Fig. 19 TEM-EDS line scans of carbon on the spent (a) m-SiO_2 and (b) Mn_3O_4 particles distributed outside the m-SiO_2 nanospheres.

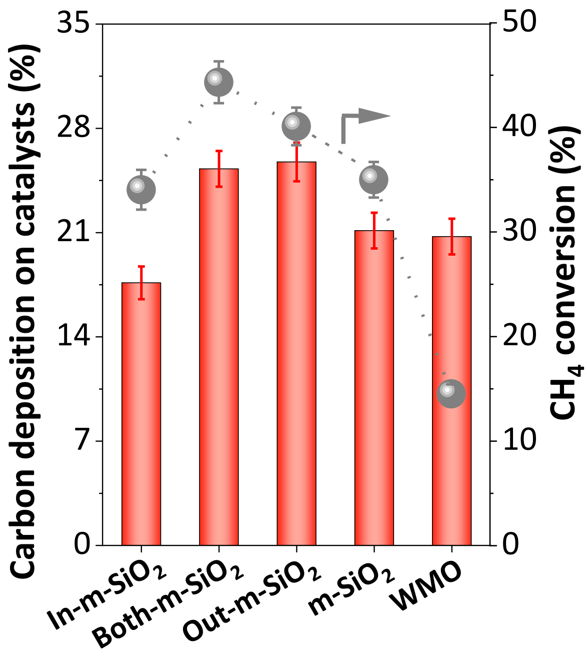

Supplementary Fig. 20 Selectivity of carbon deposited on the catalyst and methane conversion (Conditions: 1 bar, SEI 5.1 kJ L^{-1}, total flow rate 200 mL min^{-1}, discharge power 17 W, experiment duration 60 min). Error bars (standard deviation) in the figure were obtained from three sampling runs.

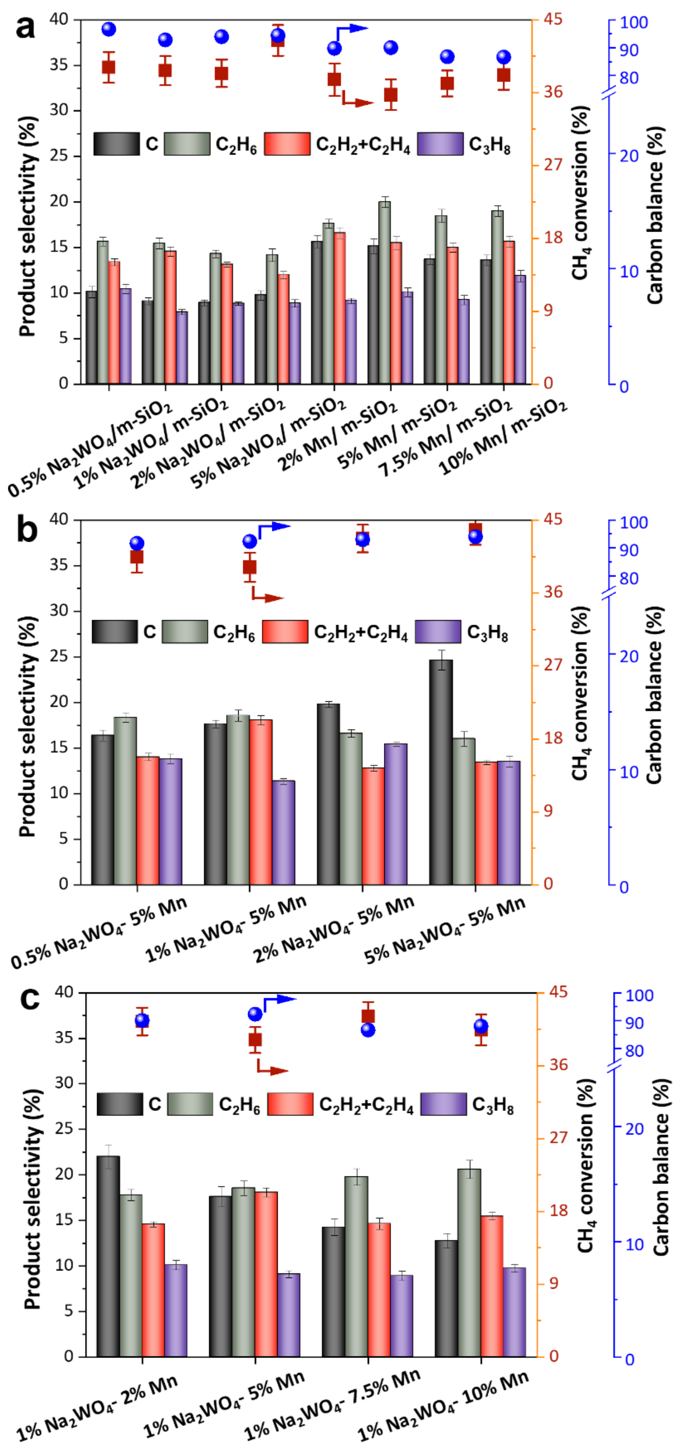

Supplementary Fig. 21 Product selectivity (C, C_2H_6, C_2H_2 and C_2H_4, and C_3H_8), CH_4 conversion and carbon balance via (a) a single metal oxide (Mn-O or W-O) supported on m-SiO_2, (b) 5% Mn with varying loadings of Na_2WO_4 on m-SiO_2 and (c) 1% Na_2WO_4 with varying loadings of Mn on m-SiO_2 (Conditions: 1 bar, SEI 5.1 kJ L^{-1}, total flow rate 200 mL min^{-1}, discharge power 17 W, experiment duration 60 min). Error bars (standard deviation) in the figure were obtained from three sampling runs.

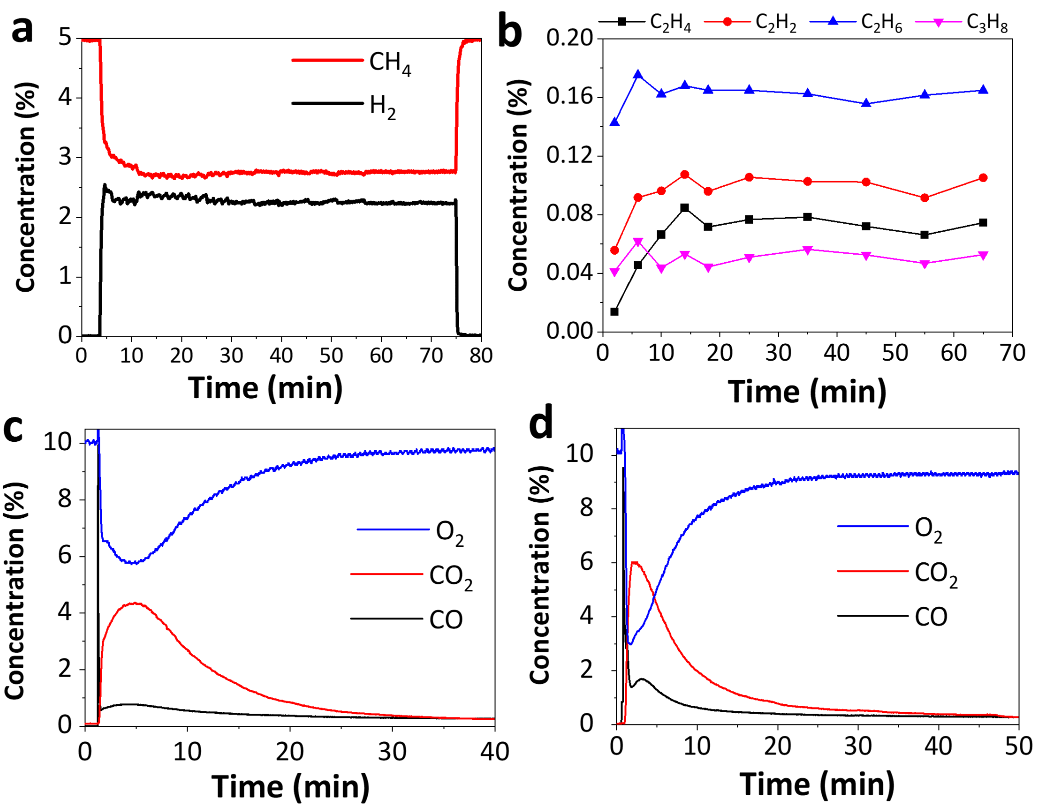

Supplementary Fig. 22 Evolution of CH_4 conversion and the concentration of C_2-C_3 hydrocarbons (C_2H_2, C_2H_4, C_2H_6 and C_3H_8) and H_2 over WMO/m-SiO_2 (a and b). Plots of O_2, CO, and CO_2 during the oxidation of carbon deposited on the (c) DBD reactor and (d) catalyst (Conditions: 1 bar, SEI 5.1 kJ L^{-1}, total flow rate 200 mL min^{-1}, discharge power 17 W, experiment duration 60 min).

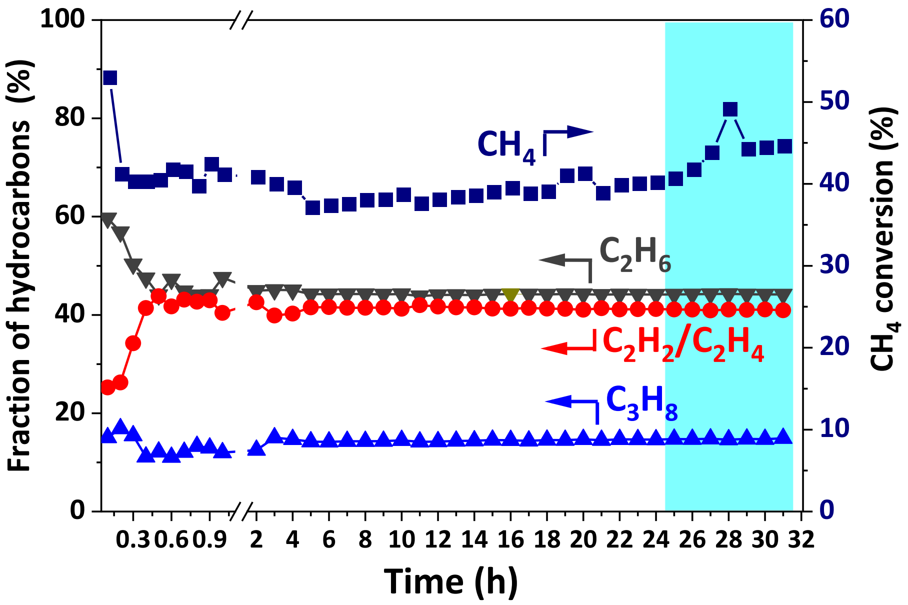

Supplementary Fig. 23 Stability test of WMO/m-SiO_2 over 32 h of reaction (Conditions: 1 bar, SEI 5.1 kJ L^{-1}, total flow rate 200 mL min^{-1}, discharge power 17 W, experiment duration 60 min).

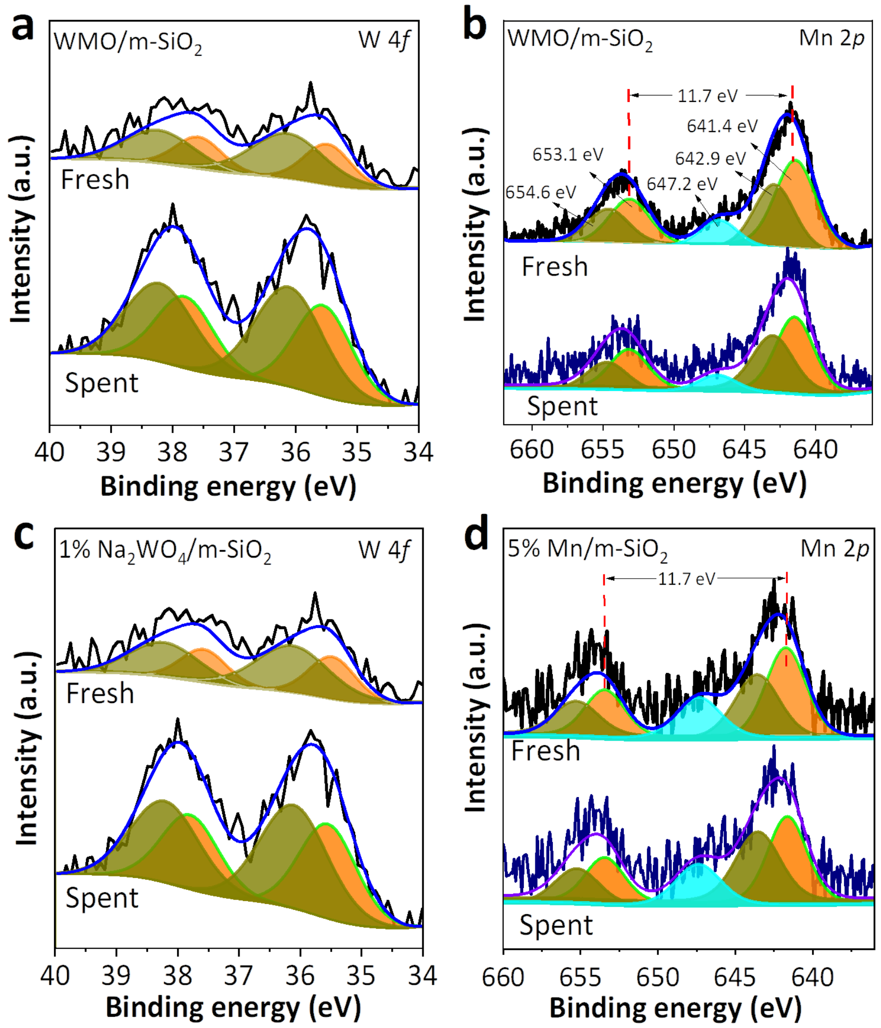

Supplementary Fig. 24 XPS spectra in the W 4*f* and Mn 2*p* regions of the fresh and spent catalysts. (a, b) WMO/m-SiO_2. (c) 1% Na_2WO_4/m-SiO_2. (d) 5% Mn/m-SiO_2.

XPS analysis of the W 4*f* spectra revealed two distinct groups of doublets in the W $4f_{7/2}$ binding energy region (Fig. 4a). These doublets correspond to the W^{6+} and W^{5+} oxidation states[17], with binding energies of approximately 36.1 eV and 35.4 eV, respectively. Notably, the surface ratio of W^{6+} to total W, defined as $W^{6+}/(W^{5+}+W^{6+})$, remained unchanged between the fresh (66.1%) and spent (67.6%) WMO /m-SiO_2 catalysts. This behavior was similar to what was observed for 5% Mn/m-SiO_2 and 1% Na_2WO_4/m-SiO_2. Similarly, the Mn 2*p* peak (Mn $2p_{3/2}$ at 641.8 eV and Mn $2p_{1/2}$ at 653.6 eV) confirmed the presence of Mn^{3+} (57.2%) and Mn^{2+} (42.8%) in Mn_3O_4 on the catalyst surface[18]. Importantly, no significant shifts in these peaks were detected for WMO/m-SiO_2 or 5% Mn/m-SiO_2 even after the reaction. Based on these observations, we conclude that the metal oxides are not fully reduced under the reaction conditions.

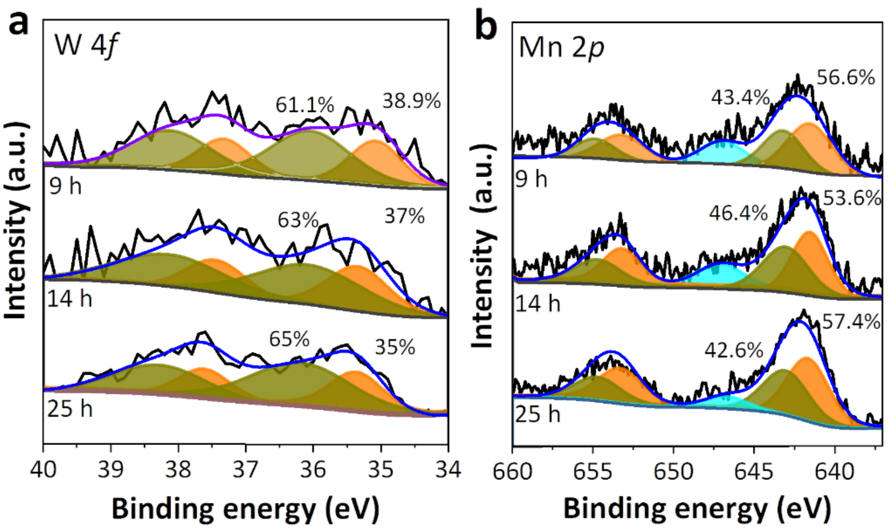

Supplementary Fig. 25 XPS spectra WMO/m-SiO_2 after reaction for 9 h, 14 h and 24 h. (a) W 4*f* region. (b) Mn 2*p* region.

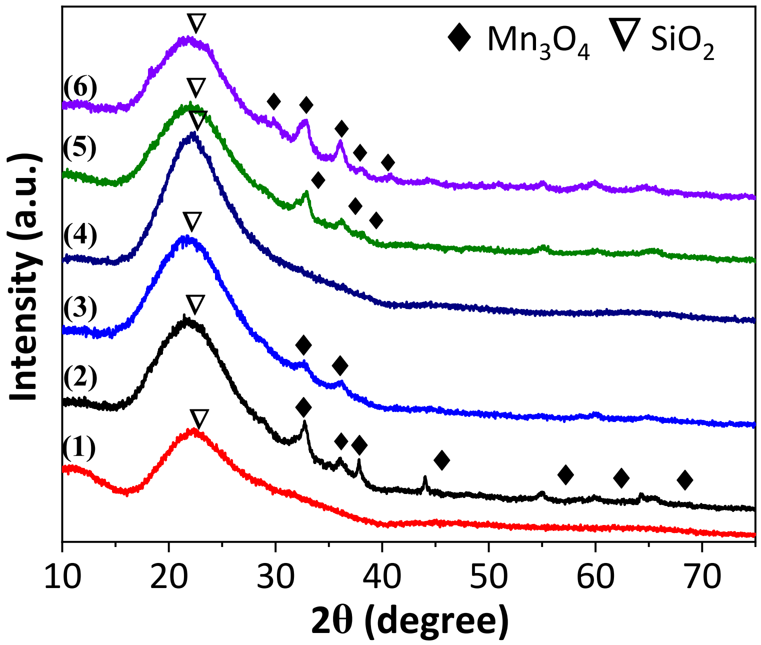

Supplementary Fig. 26 XRD patterns of fresh and spent catalysts. (1) fresh 1% Na_2WO_4/m-SiO_2. (2) fresh 5% Mn/m-SiO_2. (3) fresh WMO/m-SiO_2. (4) spent 1% Na_2WO_4/m-SiO_2. (5) spent 5% Mn/m-SiO_2. (6) spent WMO/m-SiO_2 (spent catalysts: after 24 h of reaction).

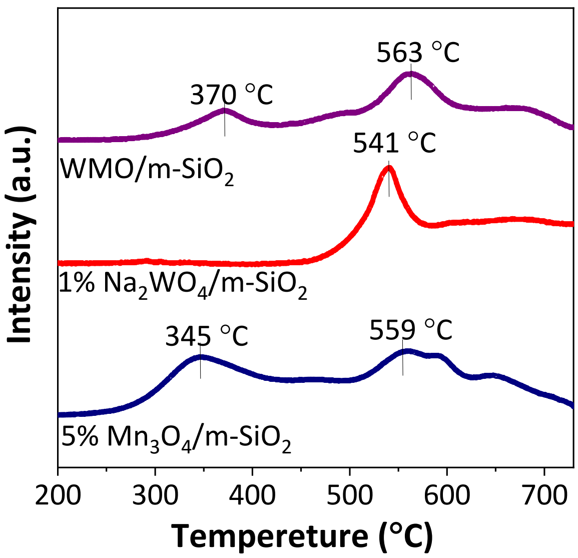

Supplementary Fig. 27 H_2-TPR profiles of WMO/m-SiO_2, 1% Na_2WO_4/m-SiO_2 and 5% Mn_3O_4/m-SiO_2.

H_2-temperature-programmed reduction (H_2-TPR) was conducted from 0 to 700 °C at a heating rate of 10 °C/min. The results revealed two primary reduction peaks for WMO/m-SiO_2. The first peak, positioned at approximately 370 °C, corresponds to the reduction of Mn_3O_4. The second peak at approximately 563 °C indicates the simultaneous reduction of both Mn_3O_4 and Na_2WO_4. In this case, the H_2 pretreatment conducted at 450 °C primarily promoted the reduction of Mn_3O_4, as evidenced by the absence of any noticeable change in the valence state of tungsten (W) in the catalyst after pretreatment, as shown in Supplementary Fig. 27.

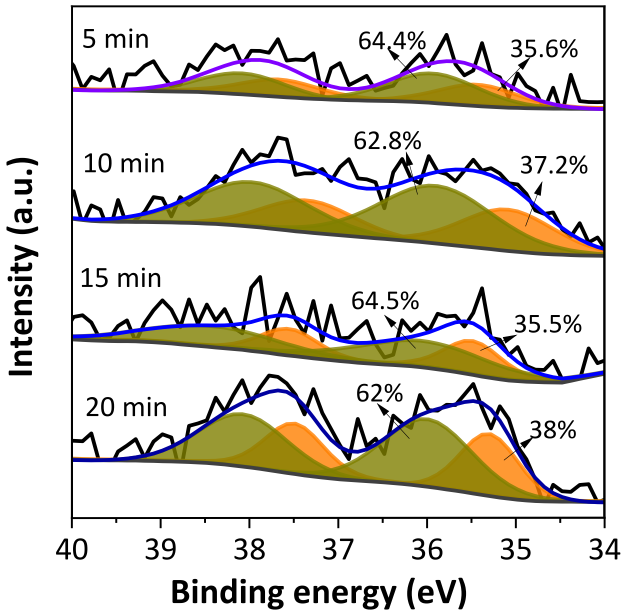

Supplementary Fig. 28 XPS spectra in the W 4*f* region of H_2 pretreated samples for 5 min, 10 min, 20 min and 25 min. The reduction temperature was maintained at 450 °C.

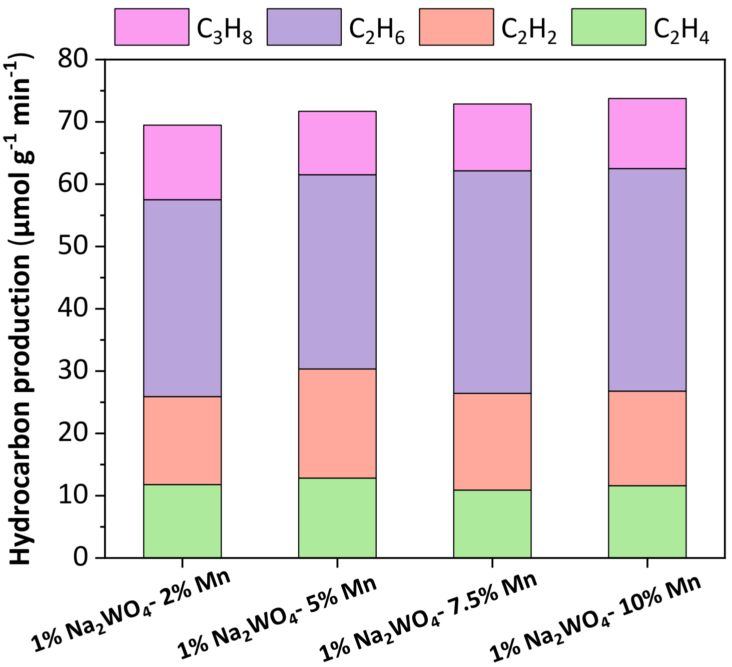

Supplementary Fig. 29 Production rates of C_2H_2, C_2H_4, C_2H_6 and C_3H_8 using 1% Na_2WO_4-β Mn_3O_4/m-SiO_2 (β = 2%, 5%, 7.5% and 10%) (Conditions: 1 bar, SEI 5.1 kJ L^{-1}, total flow rate 200 mL min^{-1}, discharge power 17 W, experiment duration 60 min).

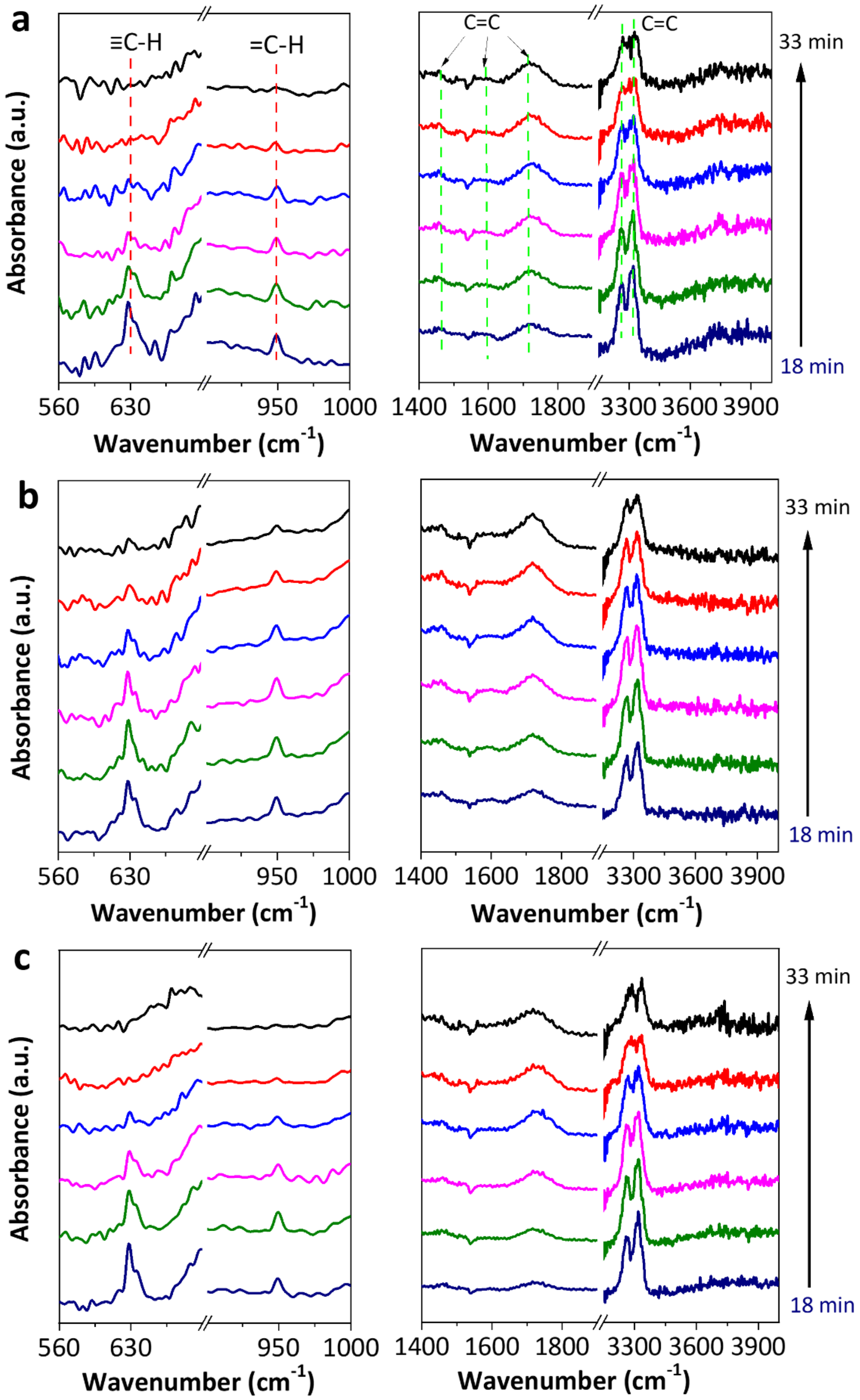

Supplementary Fig. 30 *In situ* FTIR spectra of different catalysts under plasma activation. (a) Mn_3O_4/m-SiO_2. (b) WMO/m-SiO_2. (c) Na_2WO_4/m-SiO_2.

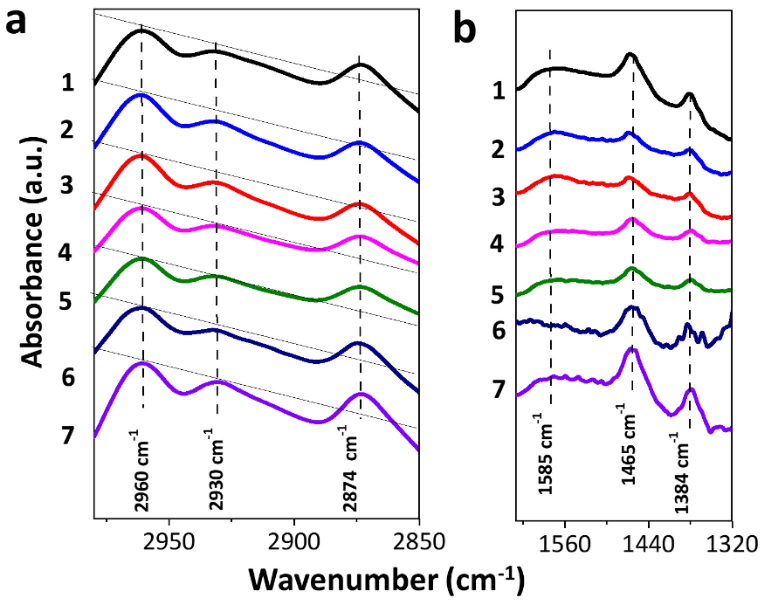

Supplementary Fig. 31 Quasi-*in situ* DRIFT spectra of different catalysts after plasma reaction in the regions of (a) 2850 - 2980 cm^{-1} and (b) 1320 - 1630 cm^{-1}. (1) 2% Mn/m-SiO_2; (2) 5% Mn/m-SiO_2; (3) 10% Mn/m-SiO_2; (4) 0.5% Na_2WO_4/m-SiO_2; (5) 1% Na_2WO_4/m-SiO_2; (6) 2% Na_2WO_4/m-SiO_2; and (7) 5% Na_2WO_4/m-SiO_2.

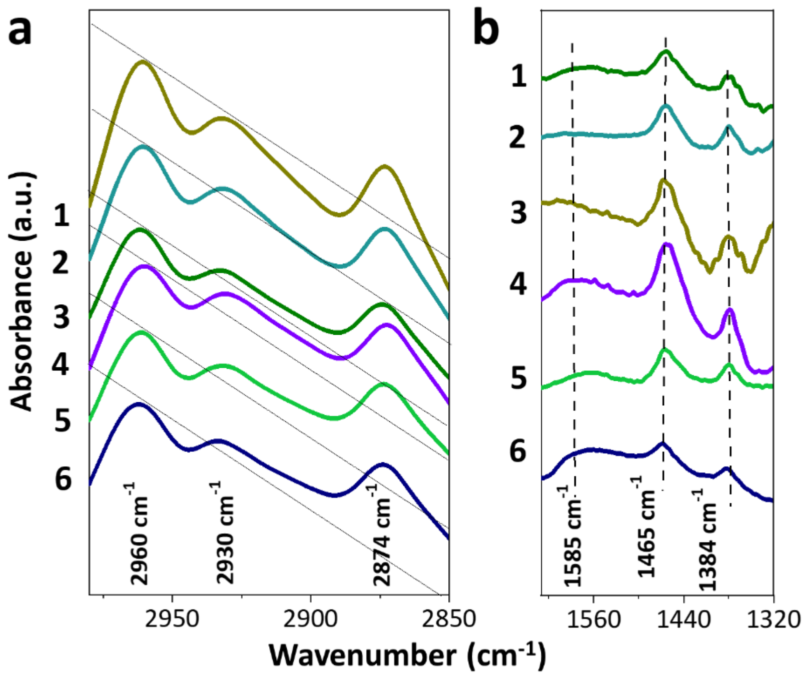

Supplementary Fig. 32 Quasi-*in situ* DRIFT spectra of different catalysts after plasma reaction in the regions of (a) 2850 - 2980 cm^{-1} and (b) 1320 to 1630 cm^{-1}. (1) 0.5% Na_2WO_4-5%Mn/m-SiO_2; (2) 1% Na_2WO_4-1%Mn/m-SiO_2; (3) 1% Na_2WO_4-2%Mn/m-SiO_2; (4) 1% Na_2WO_4-5%Mn/m-SiO_2; (5) 1% Na_2WO_4-10%Mn/m-SiO_2; (6) 5% Na_2WO_4-5%Mn/m-SiO_2.

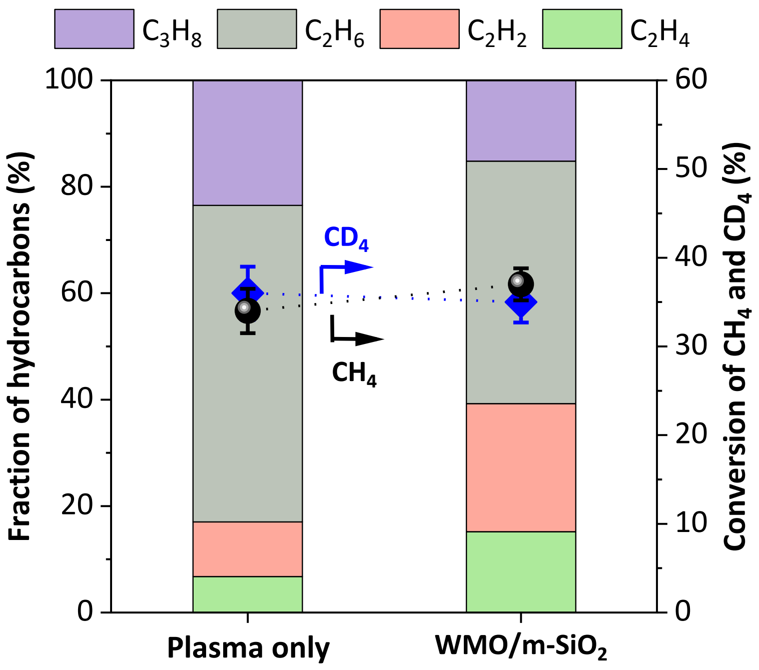

Supplementary Fig. 33 Effect of catalysts on the molar fractions of C_2-C_3 hydrocarbons and the conversion of CH_4 and CD_4 (Conditions: 1 bar, feed gas 2.5% CH_4-2.5% CD_4/Ar, SEI 5.1 kJ L^{-1}, total flow rate 200 mL min^{-1}, discharge power 17 W, experiment duration 60 min). Error bars (standard deviation) in the figure were obtained from three sampling runs.

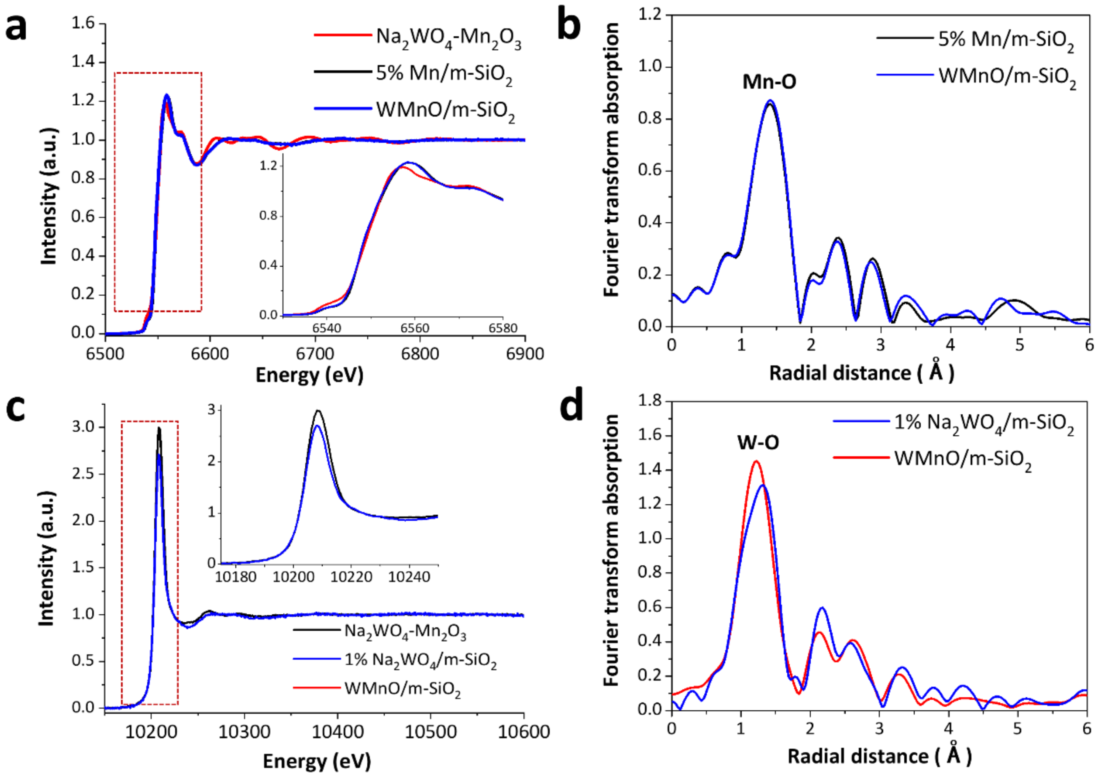

Supplementary Fig. 34 (a) Normalized X-ray absorption near-edge structure (XANES) spectra at the Mn K-edge for Na_2WO_4-Mn_2O_3, Mn_3O_4/m-SiO_2 and WMO/m-SiO_2. (b) Fourier transform (FT) XANES spectra at the Mn K-edge for Na_2WO_4-Mn_2O_3, Mn_3O_4/m-SiO_2 and WMO/m-SiO_2. (c) XANES spectra at the W K-edge for Na_2WO_4-Mn_2O_3, Na_2WO_4/m-SiO_2 and WMO/m-SiO_2. (d) FT XANES spectra at the W K-edge for Na_2WO_4-Mn_2O_3, Na_2WO_4/m-SiO_2 and WMO/m-SiO_2.

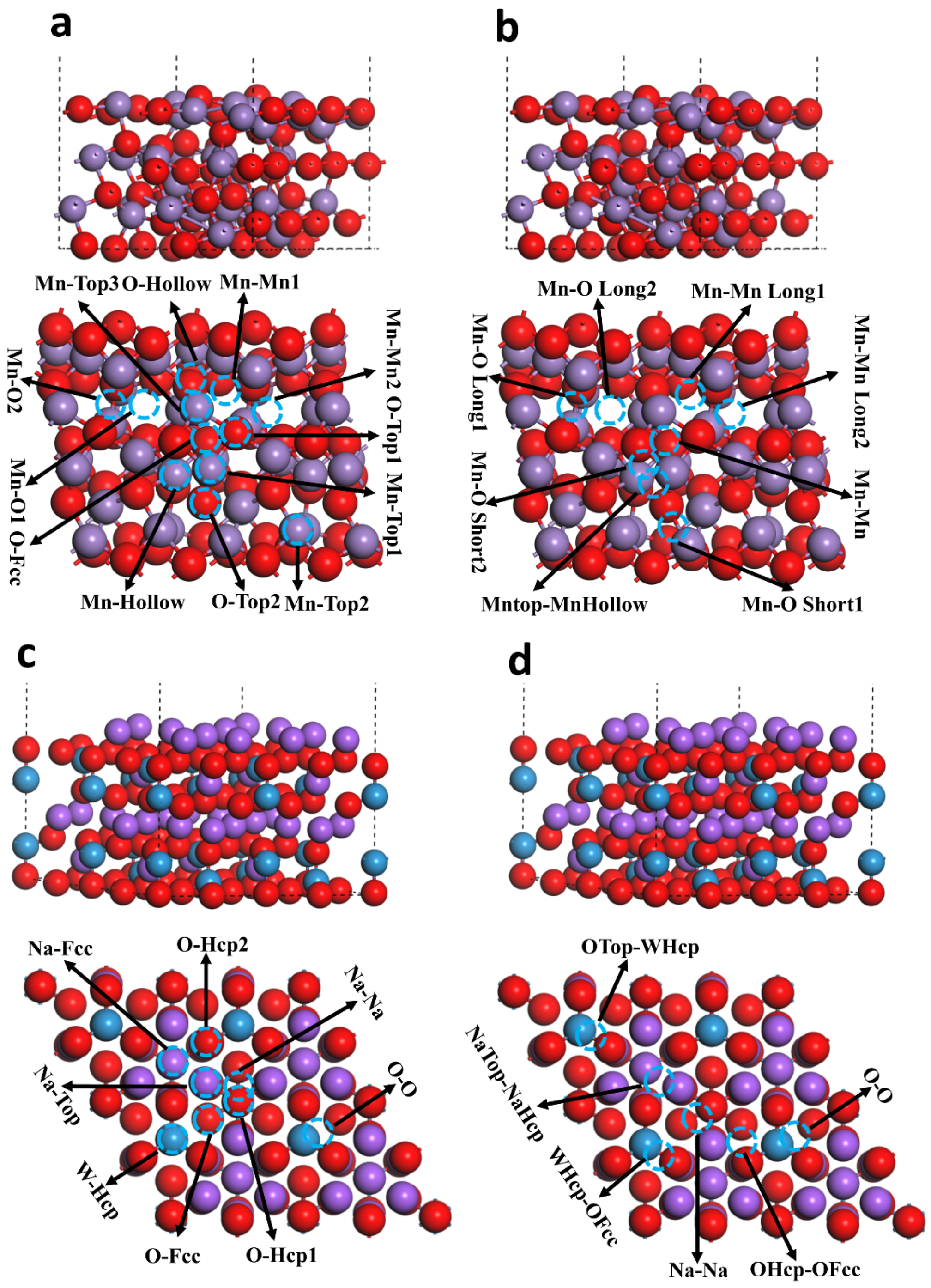

Supplementary Fig. 35 DFT-optimized structure, front view and top view. (a) Various possible adsorption sites for CH_x (x = 4, 3, 2, 1) adsorbed on the Mn_3O_4 (211) surface. (b) Various possible adsorption sites for C_xH_y (x = 2, y = 6, 4, 2) adsorbed on the Mn_3O_4 (211) surface. (c) Various possible adsorption sites for CH_x (x = 4, 3, 2, 1) adsorbed on the Na_2WO_4 (111) surface. (d) Various possible adsorption sites for C_xH_y (x = 2, y = 6, 4, 2) adsorbed on the Na_2WO_4 (111) surface. Element color coding: Mn (slate blue), O (red), Na (purple), W (dark blue).

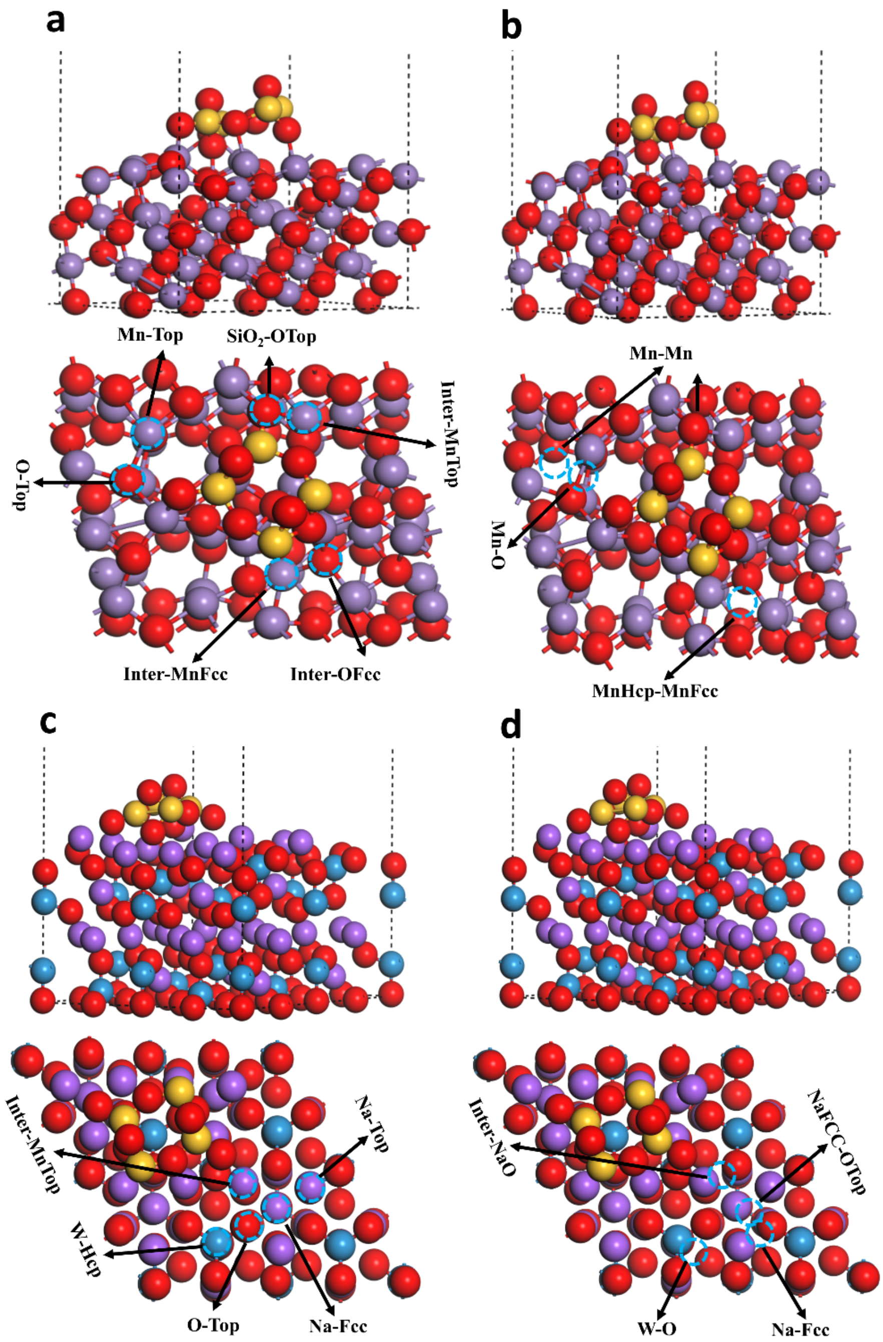

Supplementary Fig. 36 DFT-optimized structure, front view and top view. (a) Various possible adsorption sites for CH_x (x = 4, 3, 2, 1) adsorbed on the SiO_2/Mn_3O_4 (211) surface. (b) Various possible adsorption sites for C_xH_y (x = 2, y = 6, 4, 2) adsorbed on the SiO_2/Mn_3O_4 (211) surface. (c) Various possible adsorption sites for CH_x (x = 4, 3, 2, 1) adsorbed on the SiO_2/Na_2WO_4 (111) surface. (d) Various possible adsorption sites for C_xH_y (x = 2, y = 6, 4, 2) adsorbed on the SiO_2/Na_2WO_4 (111) surface. Element color coding: Mn (slate blue), O (red), Na (purple), Si (yellow), W (dark blue).

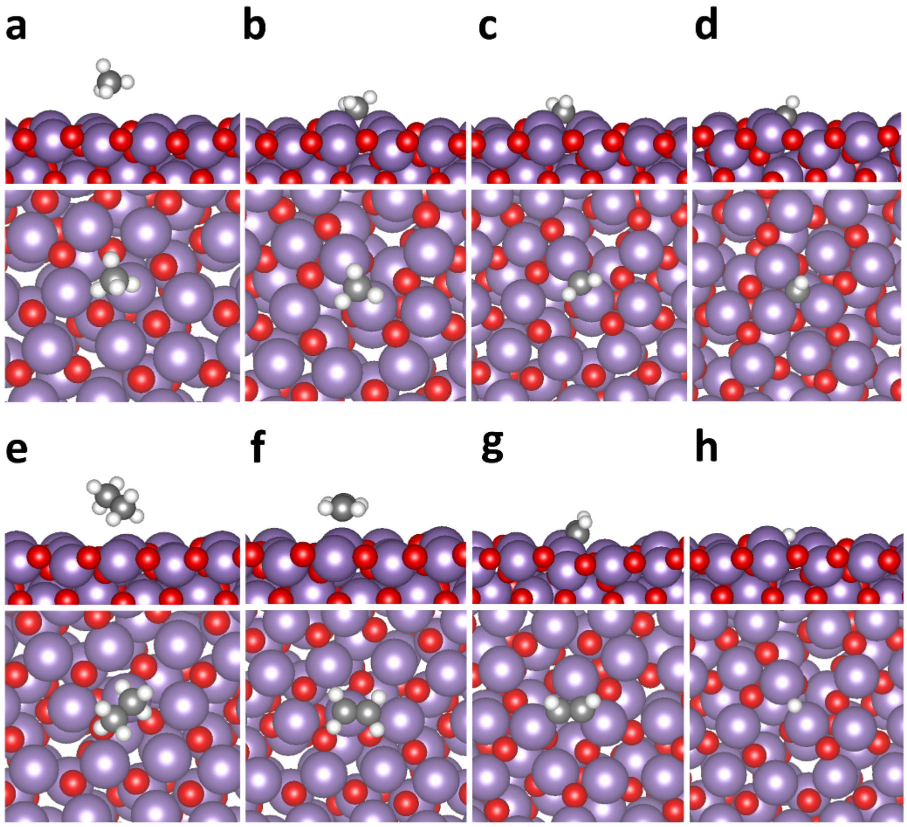

Supplementary Fig. 37 DFT-optimized geometries of intermediates in the plasma catalytic conversion of CH_4 to C_2H_2 and C_2H_4 on the Mn_3O_4 (211) surface. Top image (side view) and bottom image (top view) of (a) $^*CH_4$. (b) $^*CH_3$. (c) $^*CH_2$. (d) $^*CH$. (e) $^*C_2H_6$. (f) $^*C_2H_4$. (g) $^*C_2H_2$. (h) $^*H$. Element color coding: Mn (slate blue), O (red), C (black) and H (white).

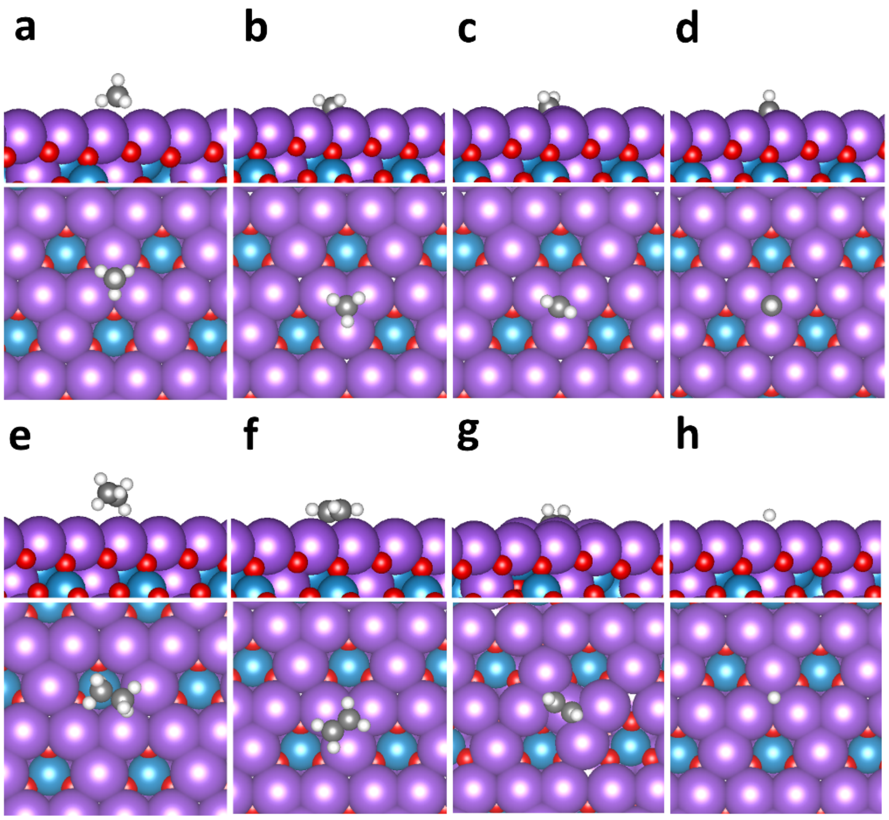

Supplementary Fig. 38 DFT-optimized geometries of intermediates for plasma catalytic conversion of CH_4 to C_2H_2 and C_2H_4 on the Na_2WO_4 (111) surface. Top image (side) and bottom image (top) views of (a) $^*CH_4$. (b) $^*CH_3$. (c) $^*CH_2$. (d) $^*CH$. (e) $^*C_2H_6$. (f) $^*C_2H_4$. (g) $^*C_2H_2$. (h) $^*H$. Element color coding: Na-purple, W-dark blue, O-red, C-black and H-white.

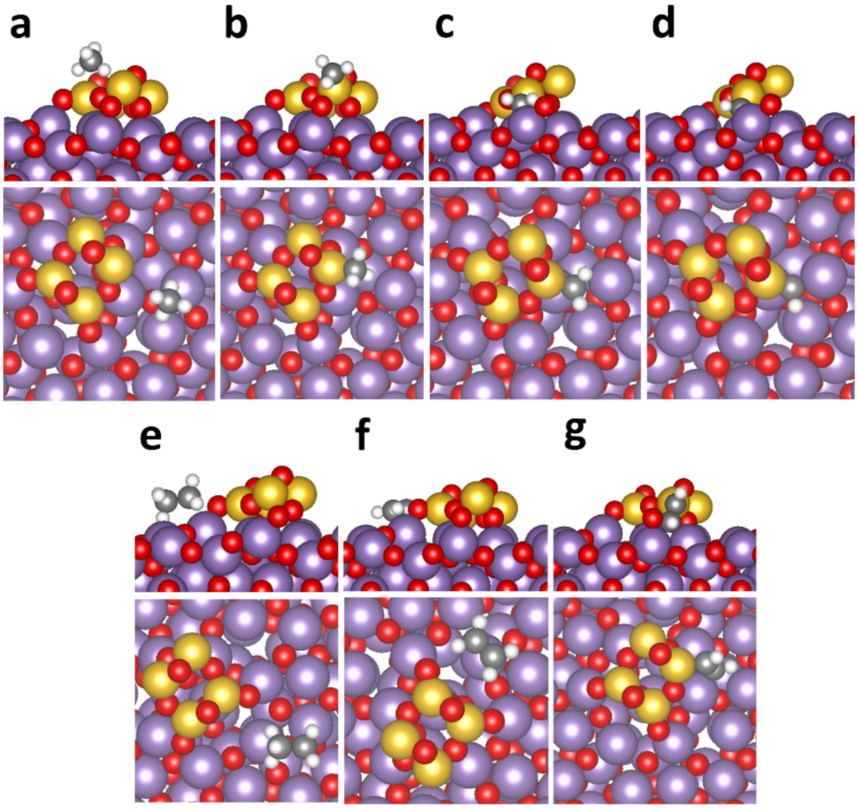

Supplementary Fig. 39 DFT-optimized geometries of intermediates in the plasma catalytic conversion of CH_4 to C_2H_2 and C_2H_4 on the SiO_2/Mn_3O_4 (211) surface. Top image (side view) and bottom image (top view) of (a) $^*CH_4$. (b) $^*CH_3$. (c) $^*CH_2$. (d) $^*CH$. (e) $^*C_2H_6$. (f) $^*C_2H_4$. (g) $^*C_2H_2$. Element color coding: Mn (slate blue), Si (yellow), O (red), C (black), H (white).

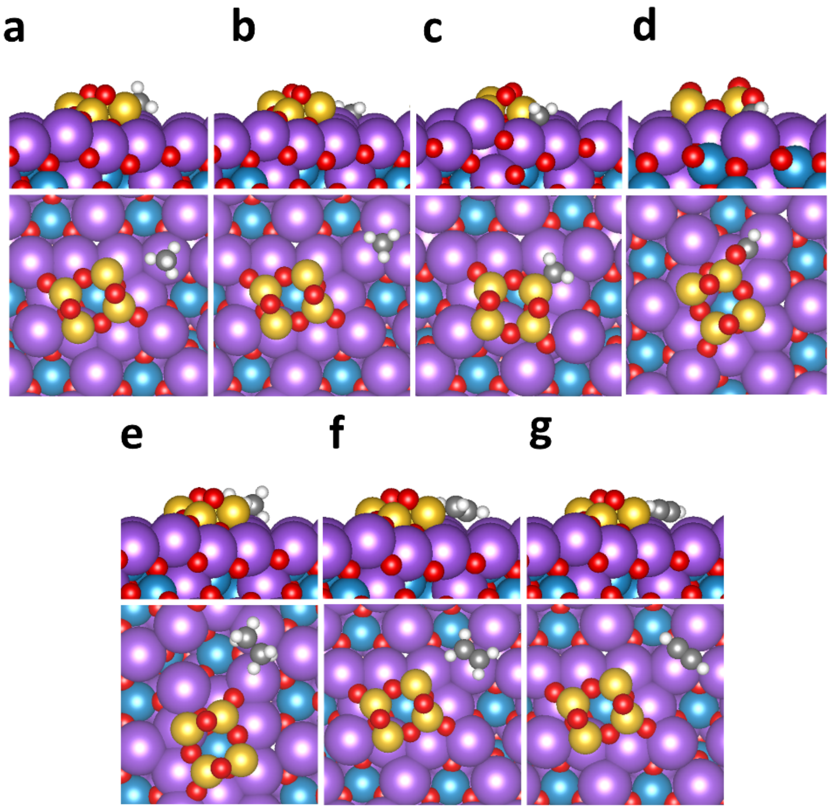

Supplementary Fig. 40 DFT-optimized geometries of intermediates in the plasma catalytic conversion of CH_4 to C_2H_2 and C_2H_4 on the SiO_2/Na_2WO_4 (111) surface. Top image (side view) and bottom image (top view) of (a) $^*CH_4$. (b) $^*CH_3$. (c) $^*CH_2$. (d) $^*CH$. (e) $^*C_2H_6$. (f) $^*C_2H_4$. (g) $^*C_2H_2$. Element color coding: Na (purple), Si (yellow), W (dark blue), O (red), C (black), H (white).

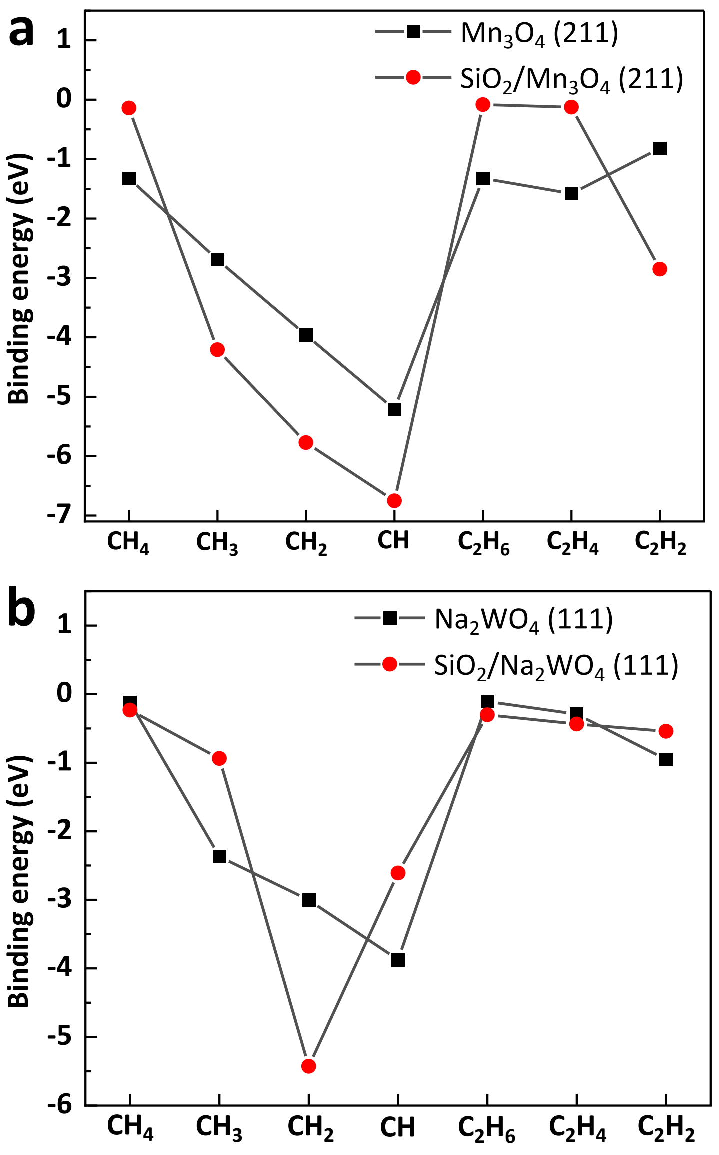

Supplementary Fig. 41 DFT-calculated binding energies of intermediates in the plasma catalytic conversion of CH_4 to C_2H_2 and C_2H_4 on the following surfaces. (a) Mn_3O_4 (211) and SiO_2/Mn_3O_4 (211). (b) Na_2WO_4 (211) and SiO_2/Na_2WO_4 (211).

3 Supplementary Tables

Supplementary Table 1 Specific surface area and total pore volume of the samples.

	BET (m^2 g^{-1})	Total pore volume (cm^3 g^{-1})
m-SiO_2	240	0.39
ZMS-5	222	0.18
SiO_2	4.3	0.01
WMO/m-SiO_2	67	0.14
WMO/ZMS-5	226	0.16
WMO/SiO_2	10	0.02

Supplementary Table 2 Comparison of CH_4 conversion (X), the fraction of C_2H_2 and C_2H_4 within C_2-C_3 hydrocarbons (F), the selectivity of C_2H_2 and C_2H_4 (R), the energy cost for the conversion of CH_4 (EC_{CH4}) in the DBD reactor between this study and the literature.

Feed Gas	Power (W)	Samples	X (%)	F (%)	R (%)	EC_{CH4} (MJ/mol)	Source
5% CH_4/Ar	17	WMO/m-SiO_2	39	42.3	18	6.8	This work
CH_4	25	Plasma only	~25	~18.5	~15	6.7	Wang et al.[19]
CH_4	45	Plasma only	25.2	25	12	4.8	Xu et al.[20]
25% CH_4/Ar	21	Plasma only	29.2	28.6	20.1	12.1	Wang et al.[21]
CH_4	45	Plasma only	~25	~26	~13	4.8	Liu et al. [22]
9% CH_4/Ar	12	γ-Al_2O_3	~15	28.6	~13	21.7	Song et al.[23]
9% CH_4/Ar	12	α-Al_2O_3	~11	34.3	~17	29.6	
CH_4	38	Ru/TiO_2	32.2	12.4	4.9	5.3	Kim et al.[24]
CH_4	38	Ru/TiO_2	35.4	10.5	4.6	4.8	
CH_4	--	Pt/γ-Al_2O_3	11	0.3	3.9	--	Kim et al.[25]
CH_4	--	Co/-Al_2O_3	28.7	15.9	5.5	--	
CH_4	--	Pt/-Al_2O_3	25.9	4.3	2.4	--	
CH_4	50	Fe/Al_2O_3	12.1	16.7	11.4	37	Indarto et al.[26]
CH_4	35	Mn/Al_2O_3	10.4	23.9	12.4	15.1	
CH_4	35	Ru/Al_2O_3	9.54	23.4	14.9	16.4	
CH_4	35	Zeolite	7.6	22	10.6	20.6	
CH_4	50	Cu/Zeolite	15.7	26.7	13.8	14.26	
CH_4	50	Ni/Zeolite	21.8	26.8	15.5	20.5	
CH_4/CO_2	40	Plasma only	25	21.8	12	8.6	Tu et al.[27]
CH_4/CO_2	50	Ni/Al_2O_3	31.4	14.6	12	8.6	
CH_4/O_2/Ar	--	Ag/SiO_2	27.5	23.9	8	--	Lee et al.[28]

EC_{CH4} =discharge power/the molar amount of converted CH_4

Supplementary Table 3 The corresponding bond length (R) and bond length factor (r-factor).

Samples	Path	R	r-factor
Mn_3O_4/m-SiO_2	Mn-O	1.86	0.011
WMO/m-SiO_2	Mn-O	1.86	0.009
Na_2WO_4-Mn_3O_4	Mn-O	1.91	0.013
Na_2WO_4/m-SiO_2	W-O	1.74	0.015
WMO/m-SiO_2	W-O	1.69	0.005
Na_2WO_4-Mn_3O_4	W-O	1.72	0.016

Supplementary Table 4. DFT-calculated binding energies (BEs) of adsorbates on the Mn_3O_4 (211) surface.

Mn_3O_4 (211) surface				
Adsorbate	Bound site	Bound via	BE (eV)	Comments
1.CH_4	Mn-Hollow	C	-0.22	
	Mn-Mn1	C	-0.27	move to Mn-Mn1
	Mn-Mn2	C	/	move to Mn-Mn1
	Mn-O1	C	/	move to Mn-Mn1
	Mn-O2	C	/	move to Mn-Mn1
	Mn-Top1	C	-1.28	
	Mn-Top2	C	-1.32	
	Mn-Top3	C	-0.20	
	O-Fcc	C	-0.21	
	O-Hollow	C	-0.19	
	O-Top1	C	-0.16	
	O-Top2	C	-0.23	
2.CH_3	Mn-Hollow	C	-2.12	
	Mn-Mn1	C	-2.70	
	Mn-Mn2	C	/	move to Mn-Mn1
	Mn-O1	C	-1.85	
	Mn-O2	C	-2.70	
	Mn-top1	C	-2.27	
	Mn-Top2	C	-2.27	
	Mn-Top3	C	-2.70	
	O-Fcc	C	-2.39	
	O-Hollow	C	/	move to Mn-Mn1
	O-Top1	C	-2.26	
	O-Top2	C	-2.12	
3.CH_2	Mn-Hollow	C	-3.39	
	Mn-Mn1	C	-3.96	
	Mn-Mn2	C	/	move to Mn-Mn1
	Mn-O1	C	-3.49	
	Mn-O2	C	-3.37	
	Mn-Top1	C	/	move to Mn-Mn1
	Mn-Top2	C	/	move to Mn-Mn1
	Mn-Top3	C	/	move to Mn-Mn1
	O-Fcc	C	/	move to Mn-Mn1
	O-Hollow	C	/	move to Mn-Mn1
	O-Top1	C	/	move to Mn-Mn1
	O-Top2	C	/	move to Mn-Mn1
4.CH	Mn-Hollow	C	/	move to Mn-Mn2

	Mn-Mn1	C	-3.13	
	Mn-Mn2	C	-4.73	
	Mn-O1	C	-4.61	
	Mn-O2	C	-4.98	
	Mn-top1	C	-5.21	Move to Mn-FCC
	Mn-top2	C	-4.53	
	Mn-top3	C	-3.46	
	O-Fcc	C	-5.20	Move to Mn-FCC
	O-Hollow	C	-3.47	
	O-Top1	C	-5.20	Move to Mn-FCC
	O-Top2	C	-5.21	Move to Mn-FCC
5.C_2H_6	Mn-Mn	C, C	-1.24	
	Mn-Mn Long1	C, C	-0.29	
	Mn-Mn Long2	C, C	-0.30	
	Mn-O Long1	C, C	-0.30	
	Mn-O Long2	C, C	-0.76	
	Mn-O Short1	C, C	-0.19	
	Mn-O Short2	C, C	-1.30	
	Mntop-MnHollow	C, C	/	Move to Mn-O short 2
	O-O	C, C	/	Move to Mn-O short 2
6.C_2H_4	Mn-Mn	C, C	-1.58	Move to Mn top
	Mn-Mn Long1	C, C	-0.69	
	Mn-Mn Long2	C, C	-0.69	Move to MnMnlong
	Mn-O Long1	C, C	/	Move to MnMnlong
	Mn-O Long2	C, C	/	Move to MnMnlong
	Mn-O Short1	C, C	/	Move to MnMnlong
	Mn-O Short2	C, C	-0.79	
	Mntop-MnHollow	C, C	-0.72	
	O-O	C, C	-1.58	Move to Mn top
7.C_2H_2	Mn-Mn	C, C	6.85	
	Mn-Mn Long1	C, C	1.49	
	Mn-Mn Long2	C, C	-0.82	
	Mn-O Long1	C, C	/	Unstable
	Mn-O Long2	C, C	-0.05	
	Mn-O Short1	C, C	/	Unstable
	Mn-O Short2	C, C	/	Unstable
	Mntop-MnHollow	C, C	-0.57	
	O-O	C, C	/	Unstable
8.H	Mn-Hollow	H	-0.33	
	Mn-Mn1	H	-1.12	
	Mn-Mn2	H	0.96	

	Mn-O1	H	2.13	
	Mn-O2	H	-0.52	
	Mn-Top1	H	-0.60	
	Mn-Top2	H	-0.45	
	Mn-Top3	H	-1.12	Move to Mn-Mn1
	O-Fcc	H	/	Move to Mn-Mn2
	O-Hollow	H	-1.12	Move to Mn-Mn1
	O-Top1	H	-0.62	
	O-Top2	H	-0.60	

Supplementary Table 5. DFT-calculated BEs of adsorbates on the Na_2WO_4 (111) surface.

Na_2WO_4 (111) surface				
Adsorbate	Bound site	Bound via	BE (eV)	Comments
1.CH_4	Na-Fcc	C	-0.12	
	Na-Na	C	-0.06	
	Na-Top	C	0.08	
	O-Fcc	C	-0.07	
	O-Hcp1	C	-0.05	
	O-Hcp2	C	-0.08	
	O-O	C	0.08	
	W-Hcp	C	-0.08	
2.CH_3	Na-Fcc	C	-1.92	
	Na-Na	C	-2.30	Move to OHcp1
	Na-top	C	-2.37	Move to OHcp1
	O-Fcc	C	-2.37	Move to OHcp1
	O-Hcp1	C	-2.37	
	O-Hcp2	C	/	
	O-O	C	-2.37	Move to OHcp1
	W-Hcp	C	-1.19	
3.CH_2	Na-Fcc	C	-2.45	
	Na-Na	C	-3.02	Move to Ofcc
	Na-Top	C	-3.08	Move to Ofcc
	O-Fcc	C	-3.00	
	O-Hcp1	C	-3.03	Move to Ofcc
	O-Hcp2	C	-2.85	
	O-O	C	-3.08	Move to Ofcc
	W-Hcp	C	-2.51	
4CH	Na-Fcc	C	-3.27	
	Na-Na	C	/	Move to O-Hcp1
	Na-Top	C	/	Move to O-Hcp1
	O-Fcc	C	/	Move to O-Hcp1
	O-Hcp1	C	/	
	O-Hcp2	C	-3.17	
	O-O	C	/	Move to O-Hcp1
	W-Hcp	C	-3.16	
5.C_2H_6	Na-Na	C, C	0.16	
	NaTop-NaHcp	C, C	0.11	
	OHcp-OFcc	C, C	0.20	
	O-O	C, C	-0.10	
	OTop-WHcp	C, C	-0.11	
	WHcp-OFcc	C, C	/	Move to Otop-Whcp
6.C_2H_4	Na-Na	C, C	-0.10	

	NaTop-NaHcp	C, C	-0.19	
	OHcp-OFcc	C, C	-0.30	
	O-O	C, C	/	Move to Ohcp-OFcc
	OTop-WHcp	C, C	-0.04	
	WHcp-OFcc	C, C	-0.01	
7.C_2H_2	Na-Na	C, C	-0.94	Move to Na-Top
	NaTop-NaHcp	C, C	-0.95	Move to Na-Top
	OHcp-OFcc	C, C	-0.54	
	O-O	C, C	-0.93	Move to Na-Top
	Otop-WHcp	C, C	-0.95	Move to Na-Top
	WHcp-OFcc	C, C	-0.32	
8.H	Na-Fcc	H	-0.88	
	Na-Na	H	/	Move to Na-Fcc
	Na-Top	H	/	Move to Na-Fcc
	O-Fcc	H	-0.65	
	O-Hcp1	H	-0.38	
	O-Hcp2	H	-0.45	
	O-O	H	0.57	
	W-Hcp	H	0.32	

Supplementary Table 6. DFT-calculated BEs of adsorbates on the SiO_2/Mn_3O_4 (211) surface.

SiO_2/Mn_3O_4 (211) surface				
Adsorbate	Bound site	Bound via	BE (eV)	Comments
1.CH_4	Inter-MnFcc	C	-0.14	Move to Mn-Mn
	Inter-MnTop	C	0.41	
	Inter-OFcc	C	0.35	
	Mn-Top	C	-0.10	
	O-Top	C	-0.01	
	SiO_2-Otop	C	0.39	
2.CH_3	Inter-MnFcc	C	-4.21	Move to Si
	Inter-MnTop	C	0.36	
	Inter-OFcc	C	-0.88	Move to Mn-Top
	Mn-Top	C	-0.88	
	O-Top	C	-1.26	
	SiO_2-OTop	C	0.36	
3.CH_2	Inter-MnFcc	C	-5.77	Move to Si-Mn
	Inter-MnTop	C	-5.42	Move to Si-Mn
	Inter-OFcc	C	-5.08	Move to Si-Mn
	Mn-Top	C	-1.61	
	O-Top	C	-3.37	Move to Mn-O
	SiO_2-OTop	C	-3.56	Move to Si-O
4.CH	Inter-MnFcc	C	-6.75	Move to Si-Mn
	Inter-MnTop	C	-3.07	Move to Si-top
	Inter-OFcc	C	/	Unstable
	Mn-Top	C	-4.00	
	O-Top	C	-3.11	
	SiO_2-OTop	C	-3.79	
5.C_2H_6	MnHcp-MnFcc	C, C	0.22	
	Mn-Mn	C, C	-0.08	
	Mn-O	C, C	0.27	
6.C_2H_4	MnHcp-MnFcc	C, C	-0.12	Move to Mn-Ofcc
	Mn-Mn	C, C	-0.56	Move to Mn-top
	Mn-O	C, C	-0.62	
7.C_2H_2	MnHcp-MnFcc	C, C	-2.85	Move to Si-Mn
	Mn-Mn	C, C	-0.74	Move to Mn-top
	Mn-O	C, C	-0.51	

Supplementary Table 7. DFT-calculated binding energies (BEs) of adsorbates on the SiO_2/Na_2WO_4 (111) surface.

SiO_2/Na_2WO_4 (111) surface				
Adsorbate	Bound site	Bound via	BE (eV)	Comments
1.CH_4	Inter-Natop	C	-0.08	
	Na-Fcc	C	-0.11	
	Na-Top	C	-0.23	
	O-Top	C	-0.08	
	W-Hcp	C	-0.05	
2.CH_3	Inter-Natop	C	-0.93	Move to Na-Fcc
	Na-Fcc	C	-0.94	
	Na-Top	C	-0.96	Move to Na-Fcc
	O-Top	C	-0.97	Move to Na-Fcc
	W-Hcp	C	-0.09	
3.CH_2	Inter-Natop	C	-1.67	Move to Na-Fcc
	Na-Fcc	C	-1.68	
	Na-Top	C	-5.43	Move to Si-Na
	O-Top	C	-1.68	Move to Na-Fcc
	W-hcp	C	-0.19	Move to O-Fcc
4.CH	Inter-Natop	C	-3.33	Unstable
	Na-Fcc	C	-2.49	Move to Na-Fcc
	Na-Top	C	-2.48	Move to Na-Fcc
	O-Top	C	-1.42	
	W-Hcp	C	-2.61	Move to O-Fcc
5.C_2H_6	Inter-NaO	C, C	-0.30	
	NaFcc-OTop	C, C	-0.25	
	Na-O	C, C	-0.28	
	W-O	C, C	-0.18	
6.C_2H_4	Inter-NaO	C, C	-0.40	Move to Na-Na
	Nafcc-OTop	C, C	-0.43	Move to Na-top
	Na-O	C, C	-0.39	Move to Na-Na
	W-O	C, C	-0.44	Move to Na-top
7.C_2H_2	Inter-NaO	C, C	-0.54	
	NaFcc-OTop	C, C	-0.48	Move to Na-Na
	Na-O	C, C	-0.44	
	W-O	C, C	-0.48	Move to Na-Na

Supplementary Table 8. DFT calculated activation energy (E_a) on the Mn_3O_4 (211) and Na_2WO_4 (111) models.

Elementary reactions	Mn_3O_4 (211)	Na_2WO_4 (111)
	E_a (eV)	E_a (eV)
$^*CH_3 \rightarrow {}^*CH_2 + {}^*H$	1.93	0.97
$^*CH_2 \rightarrow {}^*CH + {}^*H$	2.02	0.69

Supplementary References

1 Zhang, X. et al. Synergy between β-Mo_2C nanorods and non-thermal plasma for selective CO_2 reduction to CO. *Chem* **6,** 3312-3328, (2020).

2 Kohn, W. & Sham, L. J. Self-consistent equations including exchange and correlation effects. *Phys. Rev.* **140,** A1133-A1138 (1965).

3 Hohenberg, P. & Kohn, W. Inhomogeneous electron gas. *Phys. Rev.* **136,** B864-B871 (1964).

4 G, K. & Furthmüller. J. Efficient iterative schemes for ab initio total-energy calculations using a plane-wave basis set. *Phys. Rev. B* **54,** 11169-11186 (1996).

5 Perdew, J. P. & Wang, Y. Pair-distribution function and its coupling-constant average for the spin-polarized electron gas. *Phys. Rev. B* **46,** 12947-12954 (1992).

6 Zheng, J. et al. First-principles study of native point defects in hafnia and zirconia. *Phys. Rev. B* **75,** 104112 (2007).

7 Zhu, Y. et al. 2D Co-doped $MnCr_2O_4$ nanosheets as efficient bifunctional cathode materials for long-life Li–O_2 batteries. *Inorg. Chem. Front.* **10,** 4252-4265 (2023).

8 Zou, S. et al. Surface coupling of methyl radicals for efficient low-temperature oxidative coupling of methane. *Chinese J. Catal.* **42,** 1117-1125 (2021).

9 Bayer, V. et al. Formation of MnO(001) on MnO(001): Surface and interface structural stability. *Phys. Rev. B* **76,** 165428-165428 (2007).

10 Abbas, S. A. et al. Spinel-type Na_2MoO_4 and Na_2WO_4 as promising optoelectronic materials: First-principle DFT calculations. *Chem. Phys.* **538,** 110902 (2020).

11 Tosoni, S. A DFT study of Mn_3O_4 Hausmannite thin films supported on coinage metals. *Appl. Surf. Sci.* **612,** 155920 (2023).

12 Xie, Z. *et al.* Interfacial active sites for CO_2 assisted selective cleavage of C–C/C–H bonds in ethane. *Chem* **6**, 2703-2716 (2020).

13 Tian, D. *et al.* DFT insight into the oxygen vacancies formation and CH_4 activation over CeO_2 surfaces modified by transition metals (Fe, Co and Ni). *Phys. Chem. Chem. Phys.* **20**, 11912-11929 (2018).

14 Liu, Y. *et al.* Transition metal nitrides as promising catalyst supports for tuning CO/H_2 syngas production from electrochemical CO_2 reduction. *Angew. Chem. Int. Ed.* **59**, 11345-11348 (2020).

15 Tian, D. *et al.* Density functional theory studies of transition metal carbides and nitrides as electrocatalysts. *Chem. Soc. Rev.* **50**, 12338-12376 (2021).

16 Chen, M. et al. DFT insights into oxygen vacancy formation and chemical looping dry reforming of methane on metal-substituted CeO_2 (111) surface. *Front. Chem. Sci. Eng.* **18**, 162-176 (2024).

17 Yang, M., Wu, K., Sun, S. & Ren, Y. Regulating oxygen defects via atomically dispersed alumina on Pt/WO_x catalyst for enhanced hydrogenolysis of glycerol to 1,3-propanediol. *Appl. Catal. B Environ.* **307**, 121207-121218 (2022).

18 Jiang, H. *et al.* An aqueous dual-Ion battery cathode of Mn_3O_4 via reversible insertion of nitrate. *Angew. Chem. Int. Ed.* **58**, 5286-5291 (2019).

19 Wang, B., Yan, W., Ge, W. & Duan, X. Methane conversion into higher hydrocarbons with dielectric barrier discharge micro-plasma reactor. *J. Energy Chem.* **22**, 876-882 (2013).

20 Chao Xu & Tu, X. Plasma-assisted methane conversion in an atmospheric pressure dielectric barrier discharge reactor. *J. Energy Chem.* **22**, 420-425 (2013).

21 Wang, B., Yan, W., Ge, W. & Duan, X. Kinetic model of the methane conversion into higher hydrocarbons with a dielectric barrier discharge microplasma reactor. *Chem. Engineer. J.* **234**, 354-360 (2013).

22 Liu, S., Mei, D., Shen, Z. & Tu, X. Nonoxidative conversion of methane in a dielectric barrier discharge reactor: prediction of reaction performance based on neural network model. *J. Phys. Chem. C* **118**, 10686-10693 (2014).

23 Song, Y., Kang, H., Lee, D., Kang, W,. & Jo, S. Direct conversion of methane with non-thermal and thermal plasmas. *Int. J. Plasma Environ. Sci. Technol.* **11**, 150-155 (2018).

24 Kim, S. S., Kwon, B. & Kim, J. Plasma catalytic methane conversion over sol–gel derived Ru/TiO_2 catalyst in a dielectric-barrier discharge reactor. *Catal. Commun.* **8**, 2204-2207 (2007).

25 Kim, S. S., Lee, H., Choi, J. W., Na, B. K. & Song, H. K. Methane conversion to higher hydrocarbons in a dielectric-barrier discharge reactor with Pt/γ-Al_2O_3 catalyst. *Catal. Commun.* **8**, 1438-1442 (2007).

26 Indarto, A., Choi, J. W., Lee, H. & Song, H. K. A Brief Catalyst Study on Direct Methane

Conversion Using a Dielectric Barrier Discharge. *J. Chinese Chem. Soc.* **54**, 823-828 (2013).

27 Tu, X. & Whitehead, J. C. Plasma-catalytic dry reforming of methane in an atmospheric dielectric barrier discharge: Understanding the synergistic effect at low temperature. *Appl. Catal. B Environ.* **125**, 439-448 (2012).

28 Lee, H., Lee, D.-H., Ha, J. M. & Kim, D. H. Plasma assisted oxidative coupling of methane (OCM) over Ag/SiO_2 and subsequent regeneration at low temperature. *Appl. Catal. A Gen.* **557**, 39-45 (2018).
